# Dimensional Evolution from a Giant Molybdenum-Red Cage-like {Mo_200_} to 1D Chains Enabling Ultrahigh Proton Conduction

**DOI:** 10.1021/jacs.6c06499

**Published:** 2026-06-23

**Authors:** Duidui Zhang, Rongqing Tang, Yubin Ma, Qixin Zhao, Qi Zheng, Yongge Wei, De-Liang Long, Leroy Cronin, Weimin Xuan

**Affiliations:** † State Key Laboratory of Advanced Fiber Materials & College of Chemistry and Chemical Engineering, 12475 Donghua University, Shanghai 201620, China; ‡ State Key Laboratory of Advanced Fiber Materials & College of Materials Science and Engineering, Donghua University, Shanghai 201620, China; § Department of Chemistry, Key Lab of Organic Optoelectronics and Molecular Engineering of Ministry of Education, 12442 Tsinghua University, Beijing 100084, China; ∥ Laboratory of Flexible Electronics Technology, Tsinghua University, Beijing 100084, China; ⊥ School of Chemistry, 3526 The University of Glasgow, Glasgow G12 8QQ, United Kingdom

## Abstract

The controlled assembly of giant molybdenum-red polyoxometalates (POMs) into high-order architectures and elucidating their structure–property relationships remain a formidable challenge. We report an unprecedented 204-nuclearity hollow nanocage, {Mo_8_}_0.5_@{Mo_200_} (**1**), featuring a host–guest architecture where a {Mo_200_} shell encapsulates a *β*-{Mo_8_} guest, together with its one-dimensional chain derivative, [{Mo_8_}_0.5_@{Mo_198_}­{Mo_8_}]_n_ (**2**), in which the *β*-{Mo_8_} clusters further act as linkers. The drum-shaped {Mo_200_} shell in **1** is constructed from classical {Mo_7_} and {Mo_4_} units, complemented by phenylphosphonate­(L)-induced novel V-shaped {Mo_5_L} and {Mo_5_L}* building blocks. Featuring highly reduced molybdenum centers and localized reducing electrons on Mo–Mo bonds, it forms a massive internal cavity (∼2.2 nm × 1.5 nm). The discovery of **1** significantly expands the structural library of rare giant molybdenum-red clusters. Intriguingly, its assembly triggers a symmetry breaking from pseudo-*D*_5d_ to *C*_2h_, shifting the architecture further away from an ideal fullerene-like topology. To accommodate the directional assembly of *β*-{Mo_8_} linkers, two equatorial {Mo_5_L}* units in **1** each detach a {Mo_1_} tail, converting the {Mo_200_} cage into a {Mo_198_} entity with open connection sites to ultimately form the 1D polymeric chain **2**. Benefiting from dense proton-conductive sites and large open windows, **1** exhibits a high conductivity of 8.28 × 10^–2^ S cm^–1^ (80 °C, 98% RH). More impressively, continuous 1D pathways in **2** boost this value to an ultrahigh 1.28 × 10^–1^ S cm^–1^, ranking among the best POM-based proton conductors. This work demonstrates rare dimensional evolution in giant POMs and establishes a new paradigm for designing high-performance solid-state proton conductors.

## Graphical abstract

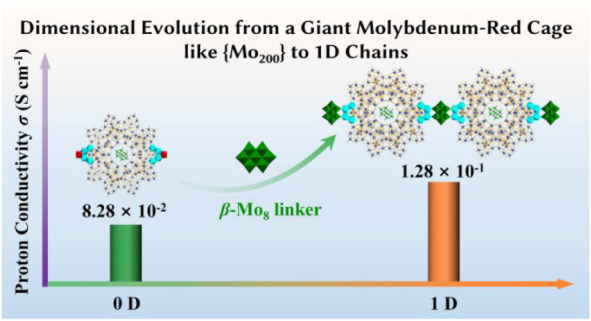

## Introduction

The biological system masterfully tunes dimensionality through polymerization of monomeric amino acids and nucleotides into chain-like peptides and nucleic acids, which play pivotal roles in life processes.^1–3^ Inspired by this natural process of converting simple monomers into complex biomacromolecules, chemists have developed efficient approaches for controlled synthesis of functional polymers from diverse organic monomers.^4–6^ Like the biosystem, synthetic polymers typically exhibit distinct or enhanced physicochemical properties compared to their monomers, leading to broad applications across various fields.^7,8^ Applying a similar principle, the emerging functional framework materials, including metal–organic frameworks (MOFs)^9–11^ and covalent-organic frameworks (COFs),^12–14^ also leverage dimensional control. With structural evolution from one dimension to higher-dimension (2D-3D) architectures, their porosity, adsorption capacity and electronic structures can be precisely tuned for gas absorption and separation, catalysis and energy storage and conversion.^15–17^ In this context, transforming nanosized inorganic clusters into extended, multidimensional structures offers a promising avenue for creating novel functional materials. These new architectures can transcend the limitations of conventional bulk inorganic solids.^18–20^

Polyoxometalates (POMs) are discrete anionic clusters constructed from metal ions and oxo ligands. Their oxygen-rich surfaces make them usable as functional building blocks (BBs) for constructing polymeric frameworks.^21–25^ Assisted by metal-oxo or metal-hydroxyl linkages, diverse 1D-3D POM-based all-inorganic frameworks can be fabricated from various clusters, including small {P_4_Mo_6_}^26^ and {V_10_},^27^ as well as classical Keggin-type {XM_12_} (X = P, Si, B etc., M = Mo, W, V),^28,29^ {V_18_},^30^ {P_5_W_30_} ^31,32^ and {P_8_W_48_},^33–35^ and even high-nuclearity {W_72_}.^36,37^ Of particular interest is the assembly of gigantic, hollow, cage-like or ring-shaped POM clusters into multidimensional aggregates. This has garnered significant attention due to their inherent porous architectures and more accessible metal and oxygen sites, which facilitate ion adsorption and transport. For example, ring-shaped {Mo_154_} and its defect derivatives {Mo_154–*x*_} (*x* denotes the missing number of Mo atoms) are well-defined monomers that readily aggregate into 1D chains, 2D layers and even 3D frameworks *via* Mo–O–Mo bridges (Scheme 1a).^38–40^ Notably, the 3D-{Mo_154_}_n_ shows proton conductivity 2–3 orders of magnitude higher than 0D-{Mo_154_} and 1D-{Mo_154_}_n_.^41^ The similar trend has also been observed in {Mo_154_}-derived W-doped {Mo_124_W_14_} system where the proton conductivity follows the sequence of 2D > 1D > 0D.^42^

**Scheme 1:**
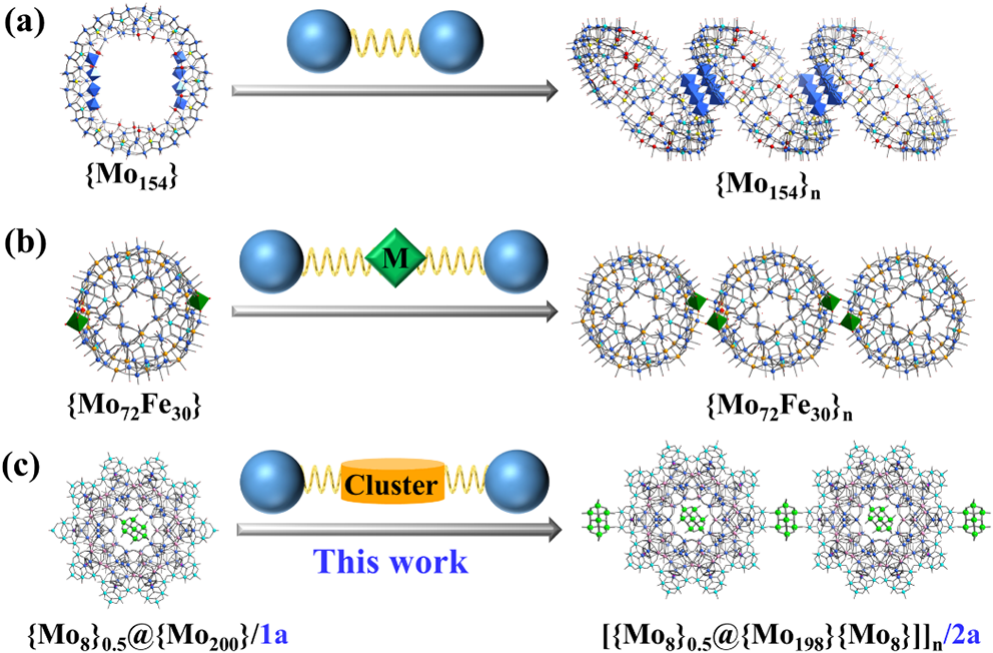
Schematic Representation of the Three Major Ways to Connect High Nuclear POM Entities; M = Transition Metal or Rare Earth Cation

Hollow cage-like clusters, in principle, provide more diffusion pathways for ion transportation compared to their ring-shaped counterpart, owing to their well-defined apertures and confined inner cavities. These distinctive features enable Keplerate-type {Mo_132_},^43^ half-closed {Ce_11_Mo_96_}^44^ and fullerene-like {Mo_240_}^45^ to achieve high proton conductivity up to 1 × 10^–1^ S cm^–1^ level generally far surpassing that of the ring-shaped counterparts. Therefore, polymerizing these clusters into high-dimensional frameworks may further extend their applications as ion transporters in fuel cell and lithium-ion battery. However, attempts to use cage-like gigantic POMs for this purpose have seen limited success. To date, the only reported success comes from the Müller’s group. They constructed 1D chains and 2D sheets by employing spherical Keplerate-type {Mo_78_Fe_24_} and {Mo_70_Fe_30_} as BBs, connected through Fe–O–Mo or Fe–O–Fe linkages (Scheme 1b).^46,47^

Advancing the exploration of such multidimensional networks necessitates the discovery of both novel cage-like, gigantic POM monomers and innovative linking strategies. Recently, we have demonstrated that employing organophosphonates as structure directing agents represents a powerful strategy to overcome these assembly challenges and enrich the structural library of gigantic POMs.^48,49^ In molybdenum blue systems, these functional ligands not only replace labile coordinated water molecules but also induce the formation of novel BBs (such as {Mo_3_L_2_} motifs and specific {Mo_1_}*, e-{Mo_2_} units). This synergistic effect enables diverse connection modes, leading to the controlled assembly of unprecedented architectures ranging from the contracted {Mo_85_} wheel, and its derived dimer {Mo_172_}^48^ to the dodecameric {Mo_16_}@{Mo_157_}^49^ host–guest framework. Moreover, capping cluster fragments are frequently *in situ* generated during the synthesis of gigantic POM clusters. This has resulted in {Mo_11_} and {Mo_30_}-capped {Mo_130_Ce_6_} and {Mo_180_}, respectively.^50^ Similarly, bicapped {Mo_248_} can also assemble from the molecular growth of {Mo_176_} by sealing both sides with a {Mo_36_} cluster.^51^ From a structural perspective, if the capping clusters can be controlled to grow on the outer surface rather than the inner rim of giant hosts, they might act as new types of linkage for structural extension. Building on these pioneering works, we speculate that combination of structure directing agent with capping cluster during synthesis may be a viable strategy to drive the formation of novel cage-like gigantic POM cluster and, consequently, the high-dimensional assembly. This approach capitalizes on the *in situ* generation of new BBs and novel connections derived from capping clusters (Scheme 1c).

Herein we report the structural evolution from hollow cage-like POM cluster **1** to its 1D chain derivative **2** (Scheme 1c). Cluster **1** features a drum-like {Mo_200_} framework that encapsulates a *β*-{Mo_8_} cluster within its ∼2.2 nm × 1.5 nm central cavity. The introduction of phenylphosphonate (L) as structural directing agent triggers the formation of novel V-shaped BB {Mo_5_L} that guides the aggregation of {Mo_200_}. Crucially, the *in situ* formation of a capping cluster of {Mo_8_} on the outer surface of a {Mo_200_}-derived node {Mo_198_} enables the structural extension of **1** into 1D chain-like structure **2**, with {Mo_8_} as a 2-connected linker (Scheme 1c). Compound **2** represents the first example of cage-based 1D chain derived from a gigantic cluster with nuclearity exceeding 200. Benefiting from the intrinsic cage-like architecture and abundant proton conductive sites both internal and external, **1** exhibits a high proton conductivity of 8.28 × 10^–2^ S cm^–1^ at 80 °C and 98% relative humidity. The reinforcement of proton transport pathway along the direction of 1D chain further enhances the proton conductivity for **2**, achieving an ultrahigh value of 1.28 × 10^–1^ S cm^–1^. This is 1.5 times higher than that of **1** and ranks among the best reported for POM-based proton conductors.

## Results and Discussion

### Synthesis and Formulas of **1** and **2**

Compound **1** was prepared by hydrothermal reaction of (NH_4_)_6_Mo_7_O_24_·4H_2_O, [N_2_H_4_]·2HCl, phenylphosphonate and NaCl at 120 °C for 3 days. Under similar conditions, increasing the stoichiometry of ammonium molybdate and replacing NaCl with KCl produced compound **2** instead. Compared with recently reported organophosphonate-directed Molybdenum Blue clusters, the synthesis of **1** employed a higher reductant-to-Mo ratio, favoring the formation of more Mo^V^ centers and Mo^V^–Mo^V^ bonded units and thereby shifting the assembly into the more highly reduced Molybdenum Red.^48,49,52^ Moreover, compared with the reported organophosphonate-functionalized {Mo_240_} cage,^53^ the higher ligand-to-Mo ratio in this work enables phenylphosphonate to act as a structure-directing agent, inducing novel V-shaped {Mo_5_L}/{Mo_5_L}* BBs.

These units redirect the assembly pathway from the ideal fullerene-like {Mo_240_}/{Mo_260_} toward a drum-like {Mo_200_}.^45,53,54^ The formulas of **1a** and **2a** were determined using a variety of characterization techniques and shown below (see Supporting Information for details):

1 $${\mathrm{Na}}_{4}{\left({\mathrm{NH}}_{4}\right)}_{42}H_{22}\left[{\left({{\mathrm{Mo}}^{\mathrm{VI}}}_{8}O_{26}\right)}_{0.5}{{\mathrm{Mo}}^{V}}_{140}{{\mathrm{Mo}}^{\mathrm{VI}}}_{60}{\left(\mathrm{OH}\right)}_{58}O_{524}{\left(C_{6}H_{5}{\mathrm{PO}}_{3}\right)}_{10}\right]{\mathrm{\cdot 250 H}}_{2}O≡{\mathrm{Na}}_{4}{\left({\mathrm{NH}}_{4}\right)}_{42}H_{22}\left\{1a\right\}{\mathrm{\cdot 250 H}}_{2}O$$

$$K_{4}{\left({\mathrm{NH}}_{4}\right)}_{44}H_{24}[{\left({{\mathrm{Mo}}^{\mathrm{VI}}}_{8}O_{26}\right)}_{0.5}{{\mathrm{Mo}}^{V}}_{140}{{\mathrm{Mo}}^{\mathrm{VI}}}_{66}{\left(\mathrm{OH}\right)}_{58}O_{544}{\left(C_{6}H_{5}{\mathrm{PO}}_{3}\right)}_{10}{]\mathrm{\cdot 260 H}}_{2}{O≡K}_{4}{\left({\mathrm{NH}}_{4}\right)}_{44}H_{24}\left\{2a\right\}{\mathrm{\cdot 260 H}}_{2}O$$ 2

### Structural Analysis of **1**

Single-crystal X-ray diffraction analysis reveals that **1** crystallizes in space group *C*ccm. Its core cluster, **1a**, adopts a hollow cage architecture, {Mo_200_}, with a *β*-{Mo_8_} cluster encapsulated in the center (Figure 1). {Mo_200_} is constructed from 10 tripodal {Mo_7_}, 20 cross-shaped {Mo_4_}, 8 {Mo_5_L} and 2 {Mo_5_L}* (L = phenylphosphonate) BBs, while {Mo_8_} guest exhibits a classical β configuration. The tripodal {Mo_7_} BBs display the same arrangement as observed in cage-like {Mo_260_} (Figure 1a).^54^ Similarly, the cross-shaped {Mo_4_} BBs are composed of a {Mo^V^_2_} dimer bridged by two edge-sharing {Mo^VI^_1_} units (Figure 1b). Five {Mo_7_} tripods interconnect with ten {Mo_4_} units to form a pentagonal {Mo_75_} macrocycle, characterized by a regular 5.5 Å aperture. This *C*_5_-symmetrical motif constitutes the bottom and upper surfaces of **1a** and is structurally related to the {Mo_75_} subunit in {Mo_260_} (Figure 1e and Figure S2).^54^ The equator of the **1a** features a well-defined cyclic {Mo_50_}, constructed through the alternating connection of 8 V-shaped {Mo_5_L} BBs and 2 {Mo_5_L}* variants in a wavy fashion (Figure 1c–d and Figure 1f). Both {Mo_5_L} and {Mo_5_L}* consist of one {Mo_1_} and two dumbbell-shaped {Mo_2_} units, bound together by one L. However, the {Mo_1_} in {Mo_5_L} adopts a tetrahedral {MoO_4_} arrangement, whereas its counterpart in {Mo_5_L}* takes an octahedral {MoO_6_} configuration (Figure 1c–d and Figure S3). Since the two {Mo_5_L}* units are arranged along diametrical direction at opposite ends of the equator, the {Mo_50_} thus displays a local symmetry of *C*_2h_ (Figure 1f).

**Figure 1:**
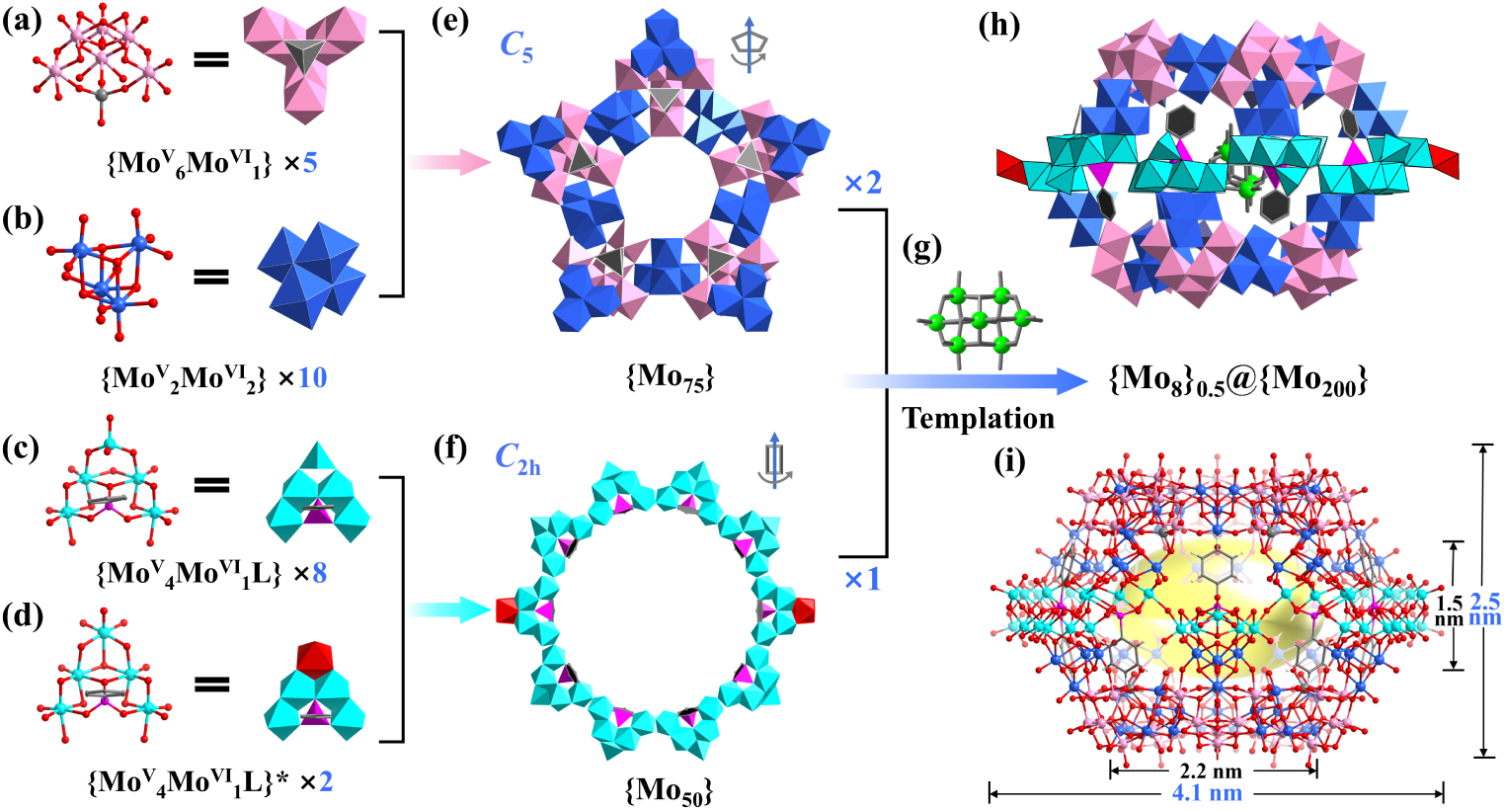
Crystal structures of cage **1a**. The connection of five {Mo_7_} (a) and ten {Mo_4_} (b) BBs to build *C*_5_-symmetric {Mo_75_} motif (e). The linkage of eight {Mo_5_L} (c) and two {Mo_5_L}* BBs (d) to construct {Mo_50_} belt (f). Two {Mo_75_} motifs, central belt {Mo_50_} and the *β*-{Mo_8_} template (g) to construct cage-shaped **1a** (h). (i) Ball-and-stick view of the structure of **1a**. Color scheme: (1) Polyhedra code: {Mo^v^_6_} in light pink and tetrahedral {Mo^VI^_1_} in gray; {Mo_4_}, blue; {Mo_5_L}, cyan; {Mo_5_L}*, two {Mo_2_} in cyan and octahedral {Mo_1_} in red; P, pink. (2) Wires code: C, gray. The phenyl ring is highlighted in dark. (3) Ball and stick code: Mo and O atoms of *β*-{Mo_8_} template are presented in bright green and gray, respectively.

The integration of two {Mo_75_} motifs with one {Mo_50_} ring, encapsulating the *β*-{Mo_8_} guest, leads to the drum-like **1a** (Figure 1h). The outer skeleton of **1a** measures ∼4.1 nm × 2.5 nm and is constructed from 200 Mo atoms, 582 O atoms and 10 phenylphosphonate ligands (Figure 1i). So far, only {Mo_368_},^55^ {Mo_248_},^51^ {Mo_240_}^45^ and its derivatives^54^ have the nuclearity exceeding 200, **1a** represents a rare example featuring with parallel giant framework. **1a** also exhibits a nanoporous inner cavity of ∼2.2 nm × 1.5 nm (Figure 1i), which is occupied by *β*-{Mo_8_}, water/protonated water molecules and counterions. Due to the wavy orientation of {Mo_50_}, one {Mo_75_} is twisted relative to the other by an angle of ∼360/10° around the approximate *C*_5_ axis (Figure 1h). This twisting mirrors that of {Mo_11_} units around approximate *C*_7_ and *C*_8_ axes in {Mo_154_} and {Mo_176_}, respectively. This arrangement clearly shows a symmetry breaking from the two ends of **1a** to its equator; specifically, the symmetry decreases from *C*_5_ to *C*_2h_ due to the coexistence of structurally inequivalent {Mo_5_L} and {Mo_5_L}* units in {Mo_50_} (Figure S4). This not only explains why **1a** adopts drum-like architecture instead of perfect fullerene-like topology seen in {Mo_240_}^45^ and {Mo_260_},^54^ but also highlights the crucial role of phenylphosphonate in directing the assembly of **1a**. Indeed, symmetry breaking is quite important for synthesizing gigantic POM clusters, as it allows the combination of two moieties with different yet compatible symmetries into a single entity, a principle that led to the discovery of hedgehog-like {Mo_368_} and hat-shaped {Mo_180_}.^50,55^

As a direct manifestation of this symmetry-breaking principle, the deviation from ideal *C*_5_-symmetry in the equator results in the overall structure of **1a** adopting pseudo *S*_10_ symmetry, which is commonly found for supramolecular assemblies built from inherent 5-fold symmetric synthons. As a result, 10 side windows are formed on the equator (Figure 1h). While these side windows exhibit comparable aperture of ∼5.4 Å to the top and bottom openings, they are partially blocked by L. Accordingly, multiple hydrogen bonds form between aromatic hydrogen and oxygen atoms on **1a** (3.454–3.688 Å) (Figure S5). In addition to the structure-directing effect of the phenylphosphonate ligands, the encapsulated *β*-{Mo_8_} core acts as an internal template. Consistent with established guest-templated assembly mechanisms in giant POMs, the {Mo_200_} shell adopts the identical *C*_2h_ symmetry to its *β*-{Mo_8_} guest (Figures S6–S7).^56^ This host–guest symmetry matching, as previously observed in our {Mo_8_}@{Mo_124_Ce_4_} systems,^57^ provides a secondary explanation for the symmetry breaking within the {Mo_200_} framework.

Bond valence sum calculations (BVSs) indicate that all the Mo centers in dumbbell-shaped {Mo_2_} adopt the oxidation state of +5, while those in the {Mo_1_} units are determined as +6 (Supplementary Table S1).^58^ The metal centers of *β*-{Mo_8_} are assumed to be Mo^VI^.^57^ Hence the overall reduction degree of **1a** reaches 68.6% and enters into the regime of Molybdenum Reds (MRs). MRs have recently emerged as a new subclass of polyoxometalate clusters characteristic of high reducing ratio (≥50%). The reported structures of this family are still relatively rare, so the discovery of **1** will further stimulate the expansion of this unique group.^59,60^

### Proposed Self-Assembly Pathways for **1a** Based on Structural Comparison with {Mo_260_}^54^

Since **1a** contain structural motifs derived from {Mo_260_},^54^ it is interesting to elucidate structure relationships between these clusters by simplifying the corresponding BBs into related nodes and linkers. To do so, the tripodal {Mo_7_} and V-shaped {Mo_5_L} are viewed as 3-connected nodes whereas cross-shaped {Mo_4_} are regarded as ditopic linkers (Figure 2c). Accordingly, {Mo_260_} is simplified as a pentagonal dodecahedron (Figure 2a). The host framework of {Mo_200_} in **1a** could be regarded as a squeezed analogue of pentagonal dodecahedron along the *C*_5_ axis by removing the ten {Mo_4_} on the belt of {Mo_260_}^54^ (the part between translucent blue planes in Figure 2a) and replacing ten 3-connected {Mo_6_} with eight {Mo_5_L} and two {Mo_5_L}* BBs, which connects with each other along the equatorial plane (Figure 2b).

**Figure 2:**
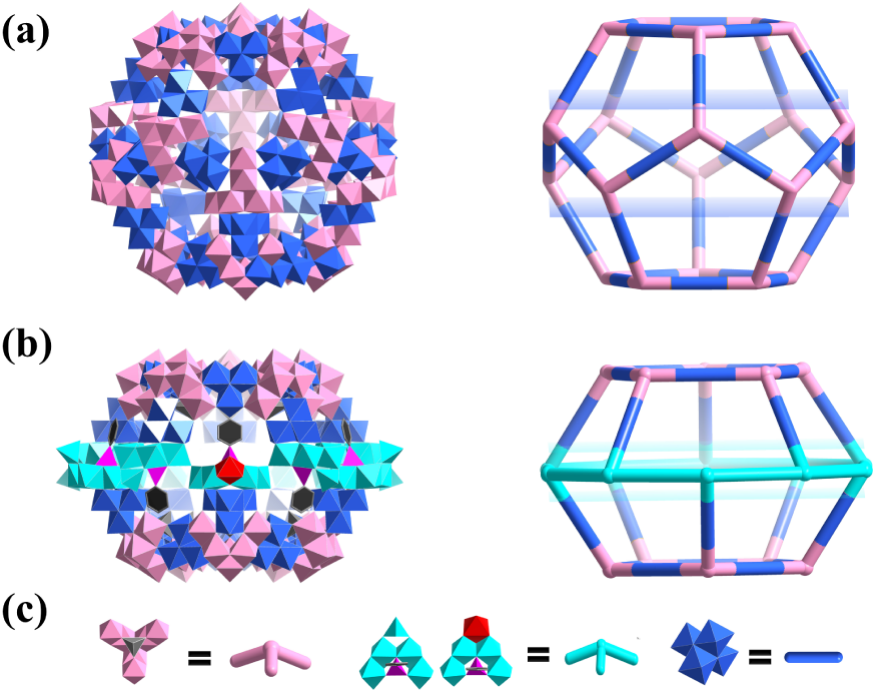
(a) Simplified polyhedral representation of {Mo_260_};^54^ (b) {Mo_200_}; (c) The {Mo_7_}, {Mo_5_L} and {Mo_5_L}* BBs are reduced as 3-connected nodes, while {Mo_4_} BB was regarded as ditopic linkers. The belt parts of {Mo_260_}^54^ and {Mo_200_} are highlighted between two translucent planes in blue and cyan, respectively.

Based on the structural analysis, cross-shaped {Mo_4_} and tripodal {Mo_7_} behave as 2-connected and 3-connected BBs. With the perfect symmetry matching, the combination of *C*_3_-symmetric {Mo_7_} and *C*_2_-symmetric {Mo_4_} will thus result in dodecahedral {Mo_260_}, as confirmed by geometric analysis in the previous study (Figure 3, up).^54^ In the presence of other BBs, the assembly will be intervened and diverges into other pathways. This is the case for cluster **1**. Assembling along *C*_5_ axis, the connection of five {Mo_7_} and ten {Mo_4_} BBs first affords {Mo_75_}, which is also observed as a pentagonal face in dodecahedral {Mo_260_}. At this stage, the further growth is stopped by the V-shaped {Mo_5_L} and {Mo_5_L}* BBs, which not only bind with two {Mo_75_} motifs to generate cage-like {Mo_200_} but also induce the symmetry breaking from two ends to belt. Meanwhile, anionic template of *β*-{Mo_8_} is also indispensable for the formation of **1a** (Figure 3, middle). This is the reason why **1a** deviates from the ideal *S*_10_ symmetry and adopts an imperfect but interesting molecular structure with a much lower symmetry.

**Figure 3:**
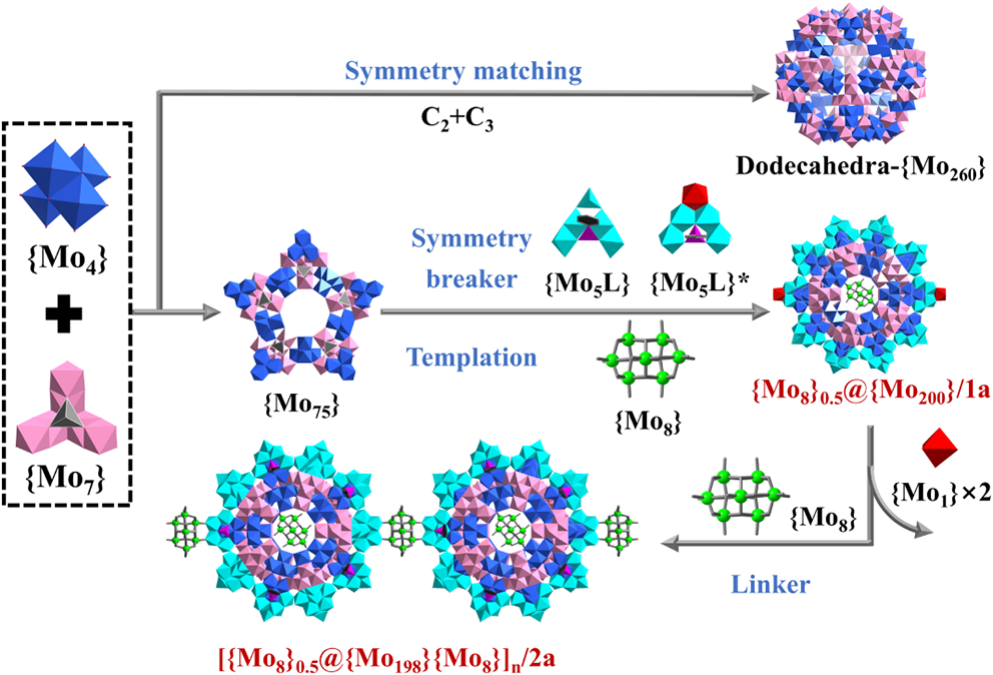
Proposed self-assembly pathways of **1** and **2**. The assembly of {Mo_4_} and {Mo_7_} BBs in the absence or the presence of capping units ({Mo_5_L} and {Mo_5_L}*), leading to the formation of {Mo_260_}^54^ (up), {Mo_8_}_0.5_@{Mo_200_} (middle) and [{Mo_8_}_0.5_@{Mo_198_}­{Mo_8_}]_n_ (bottom), respectively.

### Structural Analysis of **2**

Building upon the structural elucidation of the discrete cage **1**, we further explored its potential for higher-dimensional extension. This led to the discovery of compound **2**, which exhibits a novel 1D chain-like topological architecture (Figure 3, bottom). Structural comparison indicates that **2** is not assembled from the fully intact parent framework of **1**. Instead, the {Mo_1_} sites within the two equatorial {Mo_5_L}* BBs of **1** dissociate, generating relatively open connection sites to permit access for the *β*-{Mo_8_} linkers (Figure 4a). At the same time, the {Mo_200_} shell in **1** converts into a {Mo_198_} motif. This dissociation can be understood as an adaptive coordination adjustment process during the transformation from **1** to **2**. Acting as a 2-connected linker, the *β*-{Mo_8_} cluster achieves precise directional assembly with these exposed sites, driving the structural extension from the 0D discrete cage to the 1D polymeric chain (Figure 4b–d).

**Figure 4:**
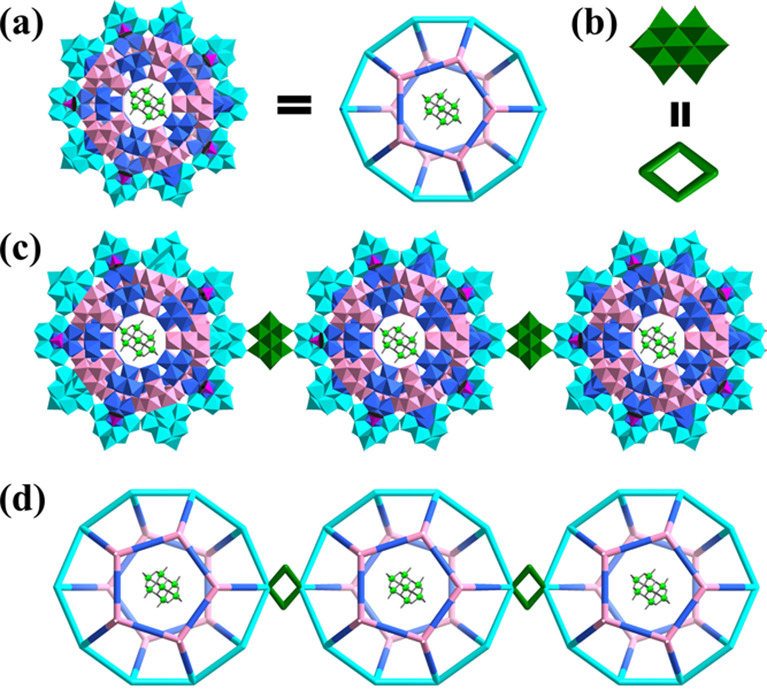
(a) Polyhedral and simplified topological representations of the monomer in **2a**; (b) The *β*-{Mo_8_} and its simplified rhombic representation; (c) Polyhedral and (d) simplified topological representations of the 1D chain structure in **2a**.

This dimensional expansion exemplifies the remarkable structural tunability inherent in {Mo_200_}-based systems, establishing them as versatile BBs for designing functional materials with tailored dimensionality. Single-crystal and powder X-ray diffraction reveal that **2** displays the same space group and unit cell parameters as **1** (*C*ccm space group). This crystallographic continuity suggests an inherent construction logic at the molecular level. Following this adaptive adjustment, multiple Mo–O–Mo bridges (bond lengths: Mo18–O92 = 2.015 Å, Mo54–O92 = 1.844 Å) are established between the compound **1** derived node {Mo_198_} and the *β*-{Mo_8_} connectors, ultimately generating a long-range ordered 1D continuous structure along the crystallographic *a*-axis. Furthermore, crystallographic analysis reveals significant disorder in the {Mo_8_} linkers. It is deduced that this disorder originates from the dynamic orientations of the *β*-{Mo_8_} templates. This conformational flexibility enables *β*-{Mo_8_} to function as an adaptive structural module, facilitating dimensional assembly through its own rotation.

The breakthrough of this work lies in **2** being the first demonstration of utilizing intact cluster units as structural linkers to construct a hollow POM-based 1D chain architecture, which fundamentally extends the theoretical framework of POM assembly. Particularly noteworthy, this cluster bridging paradigm exhibits three innovative advantages: First, the cluster-to-cluster connection strategy significantly enhances the Mo–O bond density between adjacent clusters, thereby enhancing the structural stability and the strength of the connections within the overall framework. Second, the extra M–O–M bonds enriches the ion hopping sites, making the structure highly promising for applications in ion transport and proton conductivity. Lastly, this cluster–cluster direct connection strategy breaks the space limitation of traditional POM assembly, and its successful construction provides a new paradigm for designing high-order topologies.

### Spectral Characterization of **1** and **2**

In addition to the single-crystal X-ray diffraction, the purity, composition and thermal stability of **1** and **2** were fully characterized by PXRD, TGA, FT-IR and Raman spectroscopies (Figure 5, Supplementary Table S5 and Figures S8–S12). The presence of both Mo^VI^ and Mo^V^ atoms as well as the uniform distribution of Mo, O, and P in **1** and **2** were further verified by X-ray photoelectron and energy-dispersive X-ray spectroscopies (Figure 5c and Supplementary Figures S13–14). Because **1** showed moderate solubility in water, its UV–Vis spectrum was recorded in water and displayed an absorption band around 312 nm, which is characteristic of MR clusters.^45,59^ To probe the stability of **1** in solution, **TBA-1** was prepared by cation exchange of **1** with tetrabutylammonium (TBA) in water (Supplementary Scheme S1). Uniform bright dots of **TBA-1** with sizes of 4.00 ± 0.25 nm and 3.88 ± 0.25 nm were observed in the TEM images, which are consistent with the core size of {Mo_200_} and demonstrate the stability of **1**. UV–Vis and FT-IR spectra of **1** and **TBA-1** share similar profiles and negligible degradation for 24 h (Supplementary Figures S15–17), further confirming the stability of **1** in solution and during the cation-exchange process. Furthermore, the ESI-MS spectrum of **TBA-1** in CH_3_CN displays a continuous series of multiply charged peaks from −10 to −6, and the observed *m/z* values agree well with the calculated values for intact {Mo_8_}_0.5_@{Mo_200_} species with different numbers of counterions and solvent molecules (Supplementary Figure S18 and Table S6). These results provide direct mass-spectrometric evidence for the preservation of the giant cage framework of **1** in solution.

**Figure 5:**
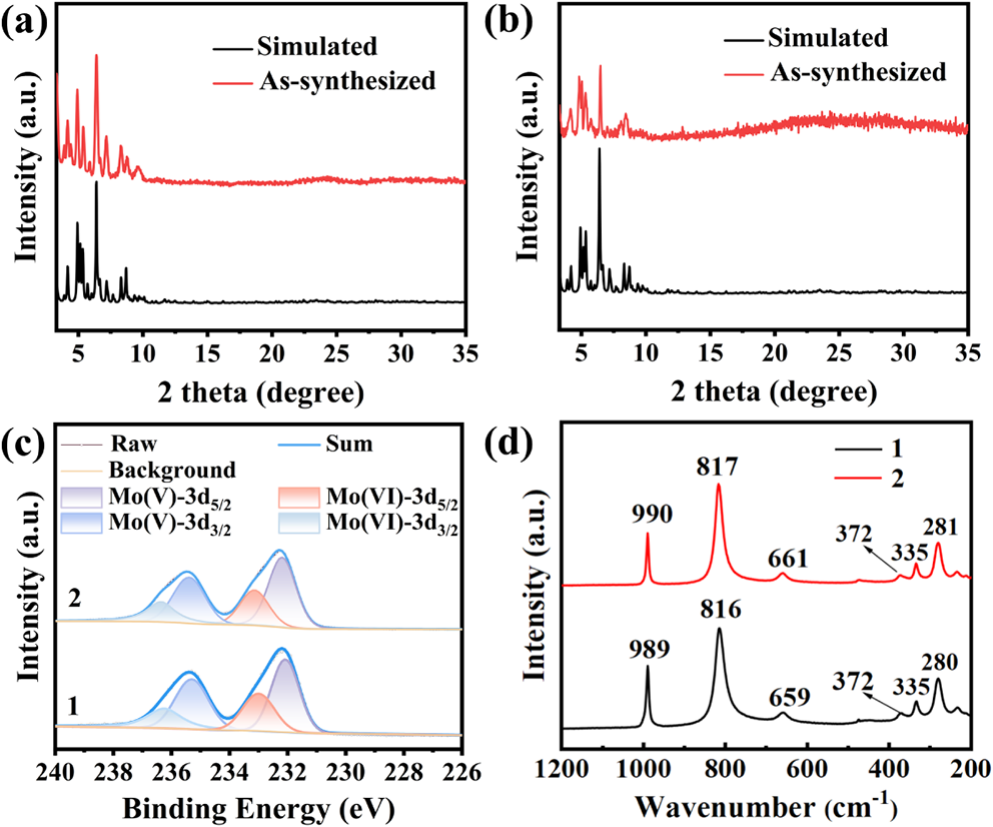
(a) and (b) PXRD patterns of **1** and **2**. (c) High resolution XPS spectra of Mo in **1** and **2**. (d) Raman spectra of **1** and **2**.

### Proton Conductivity of **1** and **2**

POMs have emerged as a premier class of solid-state proton conductors, owing to their intrinsic Brønsted acidity and oxygen-rich hydrophilic surfaces, which facilitate the formation of extensive hydrogen-bonding networks.^45^ The highly dynamic and reversible nature of H-bond formation and dissociation within these clusters effectively lowers the energy barrier for proton transport.^61,62^ Crucially, while discrete POM clusters often suffer from discontinuous pathways that create intercluster hopping bottlenecks, extending these BBs into long-range ordered frameworks provides more continuous diffusion channels, thereby maximizing the overall proton conductivity.^41,63^

The successful structural evolution from the discrete 0D cage **1** to the 1D continuous chain **2** provides an ideal platform for exploring this potential structural advantage. Consequently, the two compounds were investigated for proton conduction. The proton conductivity (σ) of **1** and **2** was determined by alternating current (AC) impedance measurement under varying relative humidity (53–98% RH) and temperature (30–80 °C). In all instances, compound **2** exhibited superior performance compared to **1** (Figure 6 and Supplementary Figures S19–S21). As observed in Nyquist plots, **2** showed an initial σ of 6.75 × 10^–4^ S cm^–1^ at 30 °C and 53% RH, which rapidly increased to 1.49 × 10^–2^ S cm^–1^ at 98% RH (Figure 6a and Table S8). A similar trend was observed for **1**, with conductivity ranging from 5.86 × 10^–6^ S cm^–1^ (53% RH) to 6.72 × 10^–3^ S cm^–1^ (98% RH). Such pronounced humidity-dependent behavior is a common feature of POM-based conductors, wherein water molecules act as vital mediators to build extensive, long-range hydrogen-bonding networks for efficient proton transport.^44,64^ This mechanism is further corroborated by the water vapor sorption isotherms of **1** and **2**, which exhibit substantial water uptake capacities (243 and 263 cm^3^ g^–1^, respectively) under high-humidity conditions (Supplementary Figure S22).

**Figure 6:**
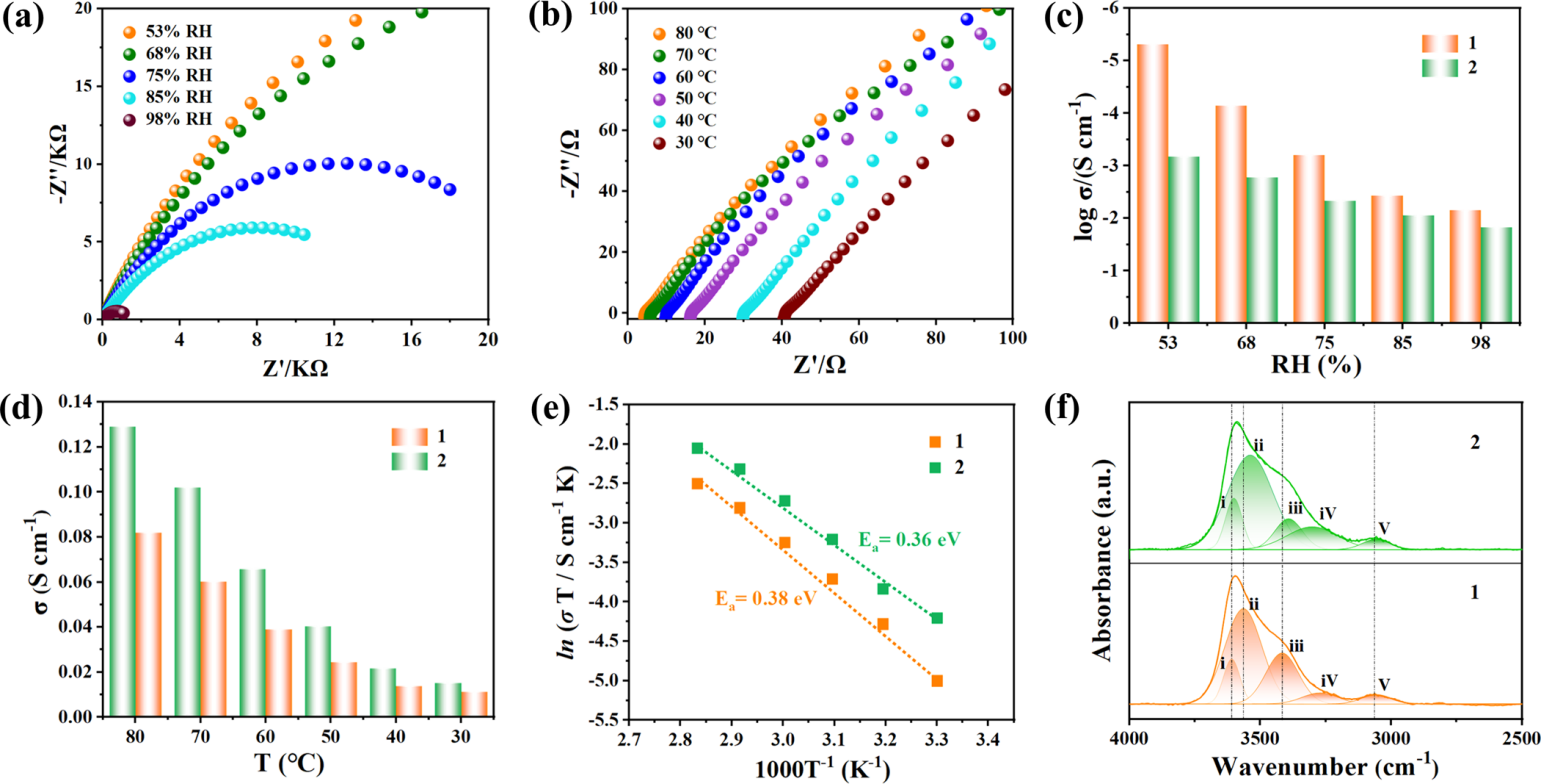
Proton conductivity at various RH and temperatures. (a) The Nyquist plots of **2** at 30 °C and various RH (53–98%). (b) Temperature-dependent proton conductivity of Compound **2** at 98% RH. (c) Comparison of the humidity-dependent proton conductivities of **1** and **2** at 30 °C. (d) Comparison of the temperature-dependent proton conductivities of **1** and **2** at 98% RH (e) Arrhenius plot of the proton conductivity of **1** and **2**. (f) The ν­(OH) region of water molecules obtained from *in situ* IR spectra of **1** and **2** under a water vapor pressure of 0.1 MPa (P/P_0_ = 0.98). The observed bands were reproduced by the sum of five Gaussian peaks.

The temperature-dependent proton transport behavior of **1** and **2** was further investigated at a fixed 98% RH. As the temperature increased from 30 to 80 °C, both compounds exhibited a steady enhancement in conductivity, reaching maximum values of 8.28 × 10^–2^ S cm^–1^ and 1.28 × 10^–1^ S cm^–1^ for **1** and **2**, respectively (Figure 6b–d, Supplementary Tables S7–S8). This enhanced conductivity at higher temperatures could be ascribed to the increased generation of H_3_O^+^ ions from the association of H_2_O and H^+^, facilitating more efficient proton transfer.^45,65^ To elucidate their proton transport behavior, the molecular structures of **1** and **2** were thoroughly analyzed. The surface oxygen sites of the clusters, lattice water molecules, and counter cations synergistically construct a complex external hydrogen-bonding network. Meanwhile, the massive inner cavity (2.2 × 1.5 nm) serves as an excellent confined container that encapsulates the *β*-{Mo_8_} template and highly enriches water molecules, thereby forming a highly ordered internal hydrogen-bonding network (Supplementary Figure S23). Crucially, these internal and external systems are seamlessly interconnected via the open windows at the top and bottom of the {Mo_200_}. Together, they weave into an extensive and robust overall hydrogen-bonded system, creating multidirectional and ordered pathways for rapid proton conduction (Supplementary Figures S24–S26). It is precisely this highly integrated and continuous hierarchical network that lays the structural foundation for their exceptional macroscopic performance. Overall, compound **2** achieves a maximum proton conductivity exceeding 10^–1^ S cm^–1^, representing one of the highest values reported for POM-based materials to date (Supplementary Table S9).^45,62,66^ Impressively, this performance rivals those of state-of-the-art crystalline proton conductors beyond the POM domain, such as benchmark MOFs,^67,68^ COFs,^69^ and HOFs.^70^ This underscores the immense potential of integrating giant cage-like POM clusters into 1D architectures to develop high efficiency solid-state proton conductors.

The underlying proton conduction mechanism was further investigated by calculating the activation energies (*E*_a_) *via* the Arrhenius equation. Both compound **1** (0.38 eV) and the 1D polymeric architecture **2** (0.36 eV) exhibit *E*_a_ values below the classical 0.4 eV threshold (Figure 6e).^44,45^ This indicates that proton transport within these giant cage clusters is governed by the Grotthuss mechanism, wherein protons migrate *via* a hopping process through the H-bonded network rather than through the diffusion of proton carriers. Crucially, the superior conductivity and lower *E*_a_ of **2** relative to **1** can be attributed to the structural extension from isolated 0D clusters into a continuous 1D polymeric architecture. In the discrete 0D system of **1**, long-range proton migration is hindered by physical gaps, relying heavily on interstitial counter cations and hydrated ions to mediate intercluster hopping across high energy barriers. Conversely, the extended Mo–O–Mo bridging bonds in **2** establish continuous pathways that overcome these barriers, enabling rapid proton transfer along the chains (Supplementary Figure S27). Furthermore, these coordination linkages significantly increase the total number of proton-hopping sites while optimizing the orientation of H-bonding sites. Consequently, the resulting 1D architecture of **2** exhibits a superior water adsorption capacity. As evidenced by the higher water uptake of **2** (263 vs 243 cm^3^ g^–1^), this enriched capacity strengthens the continuous proton-conducting network, ultimately resulting in the significantly improved transfer efficiency observed in the 1D architecture.

The role of water molecules in mediating proton conduction was investigated by analyzing the H-bonding environments of **1** and **2** using attenuated total reflection infrared (ATR-IR) spectroscopy (Figure 6f and Supplementary Figures S28–30).^71^ In the hydroxyl stretching vibration region, the broad absorption bands were deconvoluted into five characteristic peaks (Figure 6f): (i) This peak corresponds to water molecules located at the periphery of the hydrogen bonding network, typically in environments with no hydrogen bonding or weak hydrogen bonding interactions (*ca*. 3600 cm^–1^); (ii) this band represents the vibration of water molecules under weak hydrogen bonding. These water molecules form relatively weak hydrogen bonds with surrounding molecules, such as metal ions or other water molecules (*ca*. 3565 cm^–1^); (iii) this peak represents the vibration of water molecules in an environment with moderate hydrogen bonding, typically corresponding to hydrogen bonds between water molecules and oxygen atoms (*ca*. 3420 cm^–1^); (iv) this band corresponds to the overtone of the water bending vibration (δ­(HOH)), which is more sensitive to changes in hydrogen bonding but does not directly reflect variations in hydrogen bond strength (*ca*. 3260 cm^–1^); and (v) this band represents the ν­(OH) vibration of water molecules in strong hydrogen bonding environments, typically corresponding to water molecules forming strong hydrogen bonds with other water molecules or oxygen atoms within the molecule through multiple hydrogen bonds (*ca*. 3050 cm^–1^).^72^ Notably, compared to compound **1**, the overall absorption profile of compound **2** shows a noticeable shift toward lower wavenumbers, indicating the formation of a more reinforced hydrogen bonding network in the 1D structure.

To quantitatively assess the continuity of these proton conducting pathways, the relative peak areas of (i), (ii), and (v) were integrated. Peaks (i) and (ii) represent water molecules located at the expanding periphery of the hydrogen bonding network, while peak (v) represents the densely packed core of the hydrogen bonding network.^72,73^ By calculating the combined areas of these three bands, the extent of hydrogen bonding network expansion and continuity can be quantitatively evaluated. In the 1D structure, the peak areas of these three bands significantly increase, suggesting that the hydrogen bonding network in the 1D structure is denser and more continuous.

Crucially, the (iii) band represents water molecules with moderate hydrogen bonding.^71^ As demonstrated in foundational crystallographic and *in situ* IR studies, this band corresponds to water molecules forming only limited, single-point hydrogen bonds, distinguishing them from the multibonded “inner sphere” continuous network. Because these moderately bonded molecules lack the multipoint connectivity required to sustain a continuous, long-range proton transfer pathway, the peak area of the (iii) band was excluded from the quantitative calculation. The overtone of the (iv) band is less responsive to changes in hydrogen bond density, and thus, its peak area is not included in the calculation of hydrogen bond contributions. The total integrated area increased from 67% in the discrete 0D clusters of **1** to 76% in the integrated 1D chains of **2**. This quantitative enrichment confirms that the structural transition to a 1D polymeric topology facilitates a more continuous and interconnected water network, fundamentally enhancing proton transfer.^72^

To evaluate the conductivity stability, **1** and **2** were subjected to thermal cycling between 30 and 80 °C at 98% RH. The nearly coincident conductivity curves of the heating and cooling branches not only show their good tolerance to temperature fluctuations, but also verify their exceptional overall durability (Supplementary Figures S31–S32). Finally, PXRD, Raman, and IR spectroscopies were performed on the recovered samples (Supplementary Figures S33–S39). The obtained patterns and spectra exhibit no obvious differences from those of the as-synthesized materials, confirming that the host frameworks of **1** and **2** do not change during the measurements.

## Conclusion

In summary, by employing phenylphosphonate ligands as the structural director, we have successfully synthesized two giant hollow assemblies: the discrete drum-shaped cage **1** and its derived 1D polymeric analogue **2**. Structural analyses reveal that compound **2** is constructed from the compound **1**-derived nodes, achieving dimensional expansion through the adaptive coordination adjustment of the {Mo_5_L}* units and the incorporation of 2-connected {Mo_8_} clusters as linkers. Systematic proton conduction studies demonstrate remarkable performance enhancements in the 1D architecture, reaching an exceptional conductivity of 1.28 × 10^–1^ S cm^–1^ at 80 °C and 98% RH, which represents a 1.5-fold improvement over cage **1**. Notably, the successful construction of **2** demonstrates a novel paradigm of utilizing intact cluster units as linkers to bridge giant POM-based assemblies, effectively overcoming spatial limitations and optimizing transport efficiency by bridging physical gaps and minimizing hopping energy barriers. Impressively, this work significantly expands the rarely documented structural library of the MR family. It also highlights that the higher-order modular assembly of giant POM building blocks into multidimensional frameworks provides a robust blueprint for the rational design of advanced functional materials based on crystalline molecular metal oxides. Ultimately, the valuable insights gained here regarding dimensionality-enhanced proton conduction are expected to inspire the development of next-generation crystalline proton conductors and related energy conversion technologies.

## Supplementary Material



## Notes

Experimental section, synthetic procedure, single-crystal structure analyses, formula determination, structural analysis figures, thermogravimetric analysis, UV–Vis, FTIR spectra and Raman spectra, ESI-MS, and proton conduction of compounds **1** and **2**(PDF)

## References

[1] Mahon C. S., Fulton D. A. (2014). Mimicking nature with synthetic macromolecules capable
of recognition. Nat. Chem..

[2] Levin A., Hakala T. A., Schnaider L., Bernardes G. J., Gazit E., Knowles T. P. (2020). Biomimetic peptide
self-assembly
for functional materials. Nat. Rev. Chem..

[3] Mazo A. R., Allison
Logan S., Karimi F., Chan N. J. A., Qiu W., Duan W., Simpson N. M., Qiao G. G. (2020). Ring opening polymerization
of α-amino acids: advances in synthesis, architecture and applications
of polypeptides and their hybrids. Chem. Soc.
Rev..

[4] Kissel P., Erni R., Schweizer W. B., Rossell M. D., King B. T., Bauer T., Götzinger S., Schlüter A. D., Sakamoto J. (2012). A two-dimensional polymer prepared
by organic synthesis. Nat. Chem..

[5] Zhu Y., Romain C., Williams C. K. (2016). Sustainable
polymers from renewable
resources. Nature.

[6] Wypych, G. Handbook of polymers; Elsevier, 2022.

[7] Haque F. M., Grayson S. M. (2020). The synthesis, properties
and potential applications
of cyclic polymers. Nat. Chem..

[8] Hawker C. J., Wooley K. L. (2005). The convergence
of synthetic organic and polymer chemistries. Science.

[9] Furukawa H., Ko N., Go Y. B., Aratani N., Choi S. B., Choi E., Yazaydin A. Ö., Snurr R. Q., O’Keeffe M., Kim J. (2010). Ultrahigh porosity in metal-organic frameworks. Science.

[10] Furukawa H., Cordova K. E., O’Keeffe M., Yaghi O. M. (2013). The chemistry and
applications of metal-organic frameworks. Science.

[11] Xuan W., Zhu C., Liu Y., Cui Y. (2012). Mesoporous
metal-organic framework
materials. Chem. Soc. Rev..

[12] Ding S., Wang W. (2013). Covalent organic frameworks
(COFs): from design to applications. Chem. Soc.
Rev..

[13] Zhang W., Chen L., Dai S., Zhao C., Ma C., Wei L., Zhu M., Chong S. Y., Yang H., Liu L. (2022). Reconstructed
covalent organic frameworks. Nature.

[14] Côté A.
P., Benin A. I., Ockwig N. W., O’Keeffe M., Matzger A. J., Yaghi O. M. (2005). Porous,
crystalline, covalent organic
frameworks. Science.

[15] Rosi N. L., Eckert J., Eddaoudi M., Vodak D. T., Kim J., O’Keeffe M., Yaghi O. M. (2003). Hydrogen storage in microporous metal-organic
frameworks. Science.

[16] Gao P., Li W., Dong B., Zhang J., Bai X., Zhang J., Geng Y., Han X., Zai J., Pang J. (2025). Linker Enantiomericity
Engineering in Reticular Frameworks for Architecting Robust Materials
with Synergetic Open Metal Sites for Efficient SF_6_ Capture. Angew. Chem., Int. Ed..

[17] Jiang E., Chen D., Ying Z., Zhou J., Jarusarunchai A., Zhang X., Xiong C., Jeong K., Shin D.-M., Shang J. (2025). Zero- to One-Dimensional Transformation in a Highly
Porous Metal–Organic Framework to Enhance Physicochemical Properties. J. Am. Chem. Soc..

[18] Lian X., Chen H., Lin Y., Li X., Zheng S. (2023). Polyoxometalate-based
all-inorganic open frameworks. Coord. Chem.
Rev..

[19] Li Z., Li X., Yang T., Cai Z., Zheng S. (2017). Four-Shell Polyoxometalates
Featuring High-Nuclearity Ln_26_ Clusters: Structural Transformations
of Nanoclusters into Frameworks Triggered by Transition-Metal Ions. Angew. Chem., Int. Ed..

[20] Liu Q., Zhang Q., Shi W., Hu H., Zhuang J., Wang X. (2022). Self-assembly of polyoxometalate
clusters into two-dimensional clusterphene
structures featuring hexagonal pores. Nat. Chem..

[21] Miras H. N., Vilà-Nadal L., Cronin L. (2014). Polyoxometalate based open-frameworks
(POM-OFs). Chem. Soc. Rev..

[22] Vilà-Nadal L., Cronin L. (2017). Design and synthesis
of polyoxometalate-framework materials
from cluster precursors. Nat. Rev. Mater..

[23] Long D.-L., Tsunashima R., Cronin L. (2010). Polyoxometalates: building blocks
for functional nanoscale systems. Angew. Chem.,
Int. Ed..

[24] Guo Z., Yan Y., Chen Y., Li Y., Li X., Sun C., Zheng S. (2025). An all-inorganic three-dimensional polyoxoniobate framework with
ppb-level chemiresistive sensing for ammonia. Nat. Commun..

[25] Li Z., Lin L., Yu H., Li X., Zheng S. (2018). All-Inorganic Ionic
Porous Material Based on Giant Spherical Polyoxometalates Containing
Core-Shell K_6_@K_36_-Water Cage. Angew. Chem., Int. Ed..

[26] Xie S., Liu J., Dong L., Li S., Lan Y., Su Z. (2019). Hetero-metallic
active sites coupled with strongly reductive polyoxometalate for selective
photocatalytic CO_2_-to-CH_4_ conversion in water. Chem. Sci..

[27] Kar A., Sharma L., Kumar A., Halder A., Pradeep C. P. (2022). A facile
synthetic strategy for decavanadate and transition metal based all-inorganic
coordination polymers and insights into their electrocatalytic OER
activity. Eur. J. Inorg. Chem..

[28] Zhang Z., Sadakane M., Murayama T., Izumi S., Yasuda N., Sakaguchi N., Ueda W. (2014). Tetrahedral connection
of ε-Keggin-type
polyoxometalates to form an all-inorganic octahedral molecular sieve
with an intrinsic 3D pore system. Inorg. Chem..

[29] Liu J., Lin L., Wang G., Li L., Sun Y., Li X., Zheng S. (2020). All-inorganic open
frameworks based on gigantic four-shell Ln@W_8_@Ln_8_@(SiW_12_)_6_ clusters. Chem.
Commun..

[30] Zhou Y., Yao S., Yan J., Chen L., Wang T., Wang C., Zhang Z. (2015). Design and synthesis of purely inorganic 3D frameworks composed of
reduced vanadium clusters and manganese linkers. Dalton Trans..

[31] Chen L., Turo M. J., Gembicky M., Reinicke R. A., Schimpf A. M. (2020). Cation-Controlled
Assembly of Polyoxotungstate-Based Coordination Networks. Angew. Chem., Int. Ed..

[32] Turo M. J., Chen L., Moore C. E., Schimpf A. M. (2019). Co^2+^-linked
[NaP_5_W_30_O_110_]^14–^: A redox-active metal oxide framework with high electron density. J. Am. Chem. Soc..

[33] Mitchell S. G., Boyd T., Miras H. N., Long D.-L., Cronin L. (2011). Extended polyoxometalate
framework solids: two Mn­(II)-linked {P_8_W_48_}
network arrays. Inorg. Chem..

[34] Zhan C., Zheng Q., Long D.-L., Vilà Nadal L., Cronin L. (2019). Controlling the reactivity of the
[P_8_W_48_O_184_]^40–^inorganic
ring and its
assembly into POMZite inorganic frameworks with silver ions. Angew. Chem., Int. Ed..

[35] Mitchell S. G., Streb C., Miras H. N., Boyd T., Long D.-L., Cronin L. (2010). Face-directed self-assembly
of an electronically active
Archimedean polyoxometalate architecture. Nat.
Chem..

[36] Ritchie C., Streb C., Thiel J., Mitchell S. G., Miras H. N., Long D.-L., Boyd T., Peacock R. D., McGlone T., Cronin L. (2008). Reversible redox reactions in an extended polyoxometalate
framework solid. Angew. Chem., Int. Ed..

[37] Thiel J., Ritchie C., Streb C., Long D.-L., Cronin L. (2009). Heteroatom-controlled
kinetics of switchable polyoxometalate frameworks. J. Am. Chem. Soc..

[38] Ng M. T.-K., Bell N. L., Long D.-L., Cronin L. (2021). Facile and reproducible
electrochemical synthesis of the giant polyoxomolybdates. J. Am. Chem. Soc..

[39] Shishido S., Ozeki T. (2008). The pH dependent nuclearity
variation of {Mo_154‑x_}-type polyoxomolybdates and
tectonic effect on their aggregations. J. Am.
Chem. Soc..

[40] Guo H., He D., Long D.-L., Cronin L. (2025). Robotic exploration of amino-acid
functionalised molybdenum blue polyoxometalate nanoclusters. Chem. Commun..

[41] Wang H., Li S., Wang X., Long L., Kong X., Zheng L. (2021). Enhanced proton
conductivity of Mo_154_-based porous inorganic framework. Sci. China: Chem..

[42] Wu Y., Du J., Zhang H., Hou M., Li Q., Chen W., Shao K., Zhu B., Qin C., Wang X. (2024). Dimensional
regulation in gigantic molybdenum blue wheels featuring {(W)­Mo_5_} motifs for enhanced proton conductivity. Nano Res..

[43] Liu W., Dong L., Li R., Chen Y., Sun S., Li S., Lan Y. (2019). Different protonic species affecting proton conductivity
in hollow spherelike polyoxometalates. ACS Appl.
Mater. Interfaces.

[44] Li X., Li C., Hou M., Zhu B., Chen W., Sun C., Yuan Y., Guan W., Qin C., Shao K. (2023). Ce-mediated
molecular tailoring on gigantic polyoxometalate {Mo_132_}
into half-closed {Ce_11_Mo_96_} for high proton
conduction. Nat. Commun..

[45] Lin J., Li N., Yang S., Jia M., Liu J., Li X., An L., Tian Q., Dong L., Lan Y. (2020). Self-assembly
of giant
Mo_240_ hollow opening dodecahedra. J. Am. Chem. Soc..

[46] Müller A., Krickemeyer E., Das S. K., Kögerler P., Sarkar S., Bögge H., Schmidtmann M., Sarkar S. (2000). Linking Icosahedral, Strong Molecular
Magnets {MoFe}
to Layers—A Solid-State Reaction at Room Temperature. Angew. Chem., Int. Ed..

[47] Müller A., Das S. K., Talismanova M. O., Bögge H., Kögerler P., Schmidtmann M., Talismanov S. S., Luban M., Krickemeyer E. (2002). Paramagnetic
Keplerate “Necklaces”
Synthesized by a Novel Room-Temperature Solid-State Reaction: Controlled
Linking of Metal-Oxide-Based Nanoparticles. Angew. Chem., Int. Ed..

[48] Cheng M., Zhang D., Liu Y., Chen Z., Ma Y., Tang R., Wang K., Wang H., Long D.-L., Cronin L. (2025). Breaking the Boundary
of Gigantic Molybdenum Blue Clusters:
From Half-Closed {Mo_85_} to {Mo_172_} Dimer. CCS Chem..

[49] Cheng M., Li Y., Tang R., Ma Y., Long D.-L., Cronin L., Xuan W. (2026). Organophosphonate Ligation
Approach for the Controlled Assembly of
Gigantic Polyoxometalate Clusters. J. Am. Chem.
Soc..

[50] Xuan W., Pow R., Long D.-L., Cronin L. (2017). Exploring the Molecular Growth of
Two Gigantic Half-Closed Polyoxometalate Clusters {Mo_180_} and {Mo_130_Ce_6_}. Angew.
Chem., Int. Ed..

[51] Müller A., Shah S. Q., Bögge H., Schmidtmann M. (1999). Molecular
growth from a Mo_176_ to a Mo_248_ cluster. Nature.

[52] Zhang Y.-Y., Hou M.-J., Chen W.-C., Shan G.-G., Shao K.-Z., Sun C.-Y., Qin C., Wang X.-L., Su Z.-M. (2026). Anion-Guided
Controlled Molecular Growth of Multi-Component Giant Mo Wheel Family
for Enhanced Oxidation Catalysis. J. Am. Chem.
Soc..

[53] Wu S.-X., Yang Y., Qin C., Hou Y.-H., Wang X.-L., Su Z.-M. (2023). Organophosphate functionalized of {Mo_240_} polyoxomolybdate
dodecahedra. Tungsten.

[54] Lin J., Mei Z., Guo C., Li J., Kuang Y., Shi J., Liu J., Li X., Li S., Liu J., Lan Y. (2024). Synthesis
of Isotypic Giant Polymolybdate Cages for Efficient Photocatalytic
C-C Coupling Reactions. J. Am. Chem. Soc..

[55] Müller A., Beckmann E., Bögge H., Schmidtmann M., Dress A. (2002). Inorganic Chemistry Goes Protein Size: A Mo_368_ Nano-Hedgehog
Initiating Nanochemistry by Symmetry Breaking. Angew. Chem., Int. Ed..

[56] Miras H. N., Cooper G. J., Long D.-L., Bögge H., Müller A., Streb C., Cronin L. (2010). Unveiling
the transient
template in the self-assembly of a molecular oxide nanowheel. Science.

[57] Xuan W., Pow R., Watfa N., Zheng Q., Surman A. J., Long D.-L., Cronin L. (2019). Stereoselective
assembly of gigantic chiral molybdenum
blue wheels using lanthanide ions and amino acids. J. Am. Chem. Soc..

[58] Brown I. D., Altermatt D. (1985). Bond-valence
parameters obtained from a systematic
analysis of the inorganic crystal structure database. Acta Crystallogr. B.

[59] Ribó E. G., Bell N. L., Long D.-L., Cronin L. (2022). Engineering highly
reduced molybdenum polyoxometalates via the incorporation of d and
f block metal ions. Angew. Chem., Int. Ed..

[60] Long D.-L., Cronin L. (2025). Roadmap of exploring
self-assembly and the self-organization
of nanoscale polyoxometalate clusters. Adv.
Inorg. Chem..

[61] Mukherjee D., Saha A., Moni S., Volkmer D., Das M. C. (2025). Anhydrous
solid-state proton conduction in crystalline MOFs, COFs, HOFs, and
POMs. J. Am. Chem. Soc..

[62] Li S., Zhao Y., Knoll S., Liu R., Li G., Peng Q., Qiu P., He D., Streb C., Chen X. (2021). High proton-conductivity in covalently
linked polyoxometalate-organoboronic
acid-polymers. Angew. Chem., Int. Ed..

[63] Lai R., Zhang J., Li X., Zheng S., Yang G. (2022). Assemblies
of increasingly large ln-containing polyoxoniobates and intermolecular
aggregation-disaggregation interconversions. J. Am. Chem. Soc..

[64] Meng X., Wang H., Song S., Zhang H. (2017). Proton-conducting crystalline
porous materials. Chem. Soc. Rev..

[65] Chand S., Elahi S. M., Pal A., Das M. C. (2019). Metal-organic frameworks
and other crystalline materials for ultrahigh superprotonic conductivities
of 10^–2^ S cm^–1^ or higher. Chem. - Eur. J..

[66] Saha A., Sahoo R., Pal S. C., Uchida S., Das M. C. (2025). Polyoxometalates
(POMs) as proton conductors. ACS Energy Lett..

[67] Yang F., Xu G., Dou Y., Wang B., Zhang H., Wu H., Zhou W., Li J., Chen B. (2017). A flexible metal-organic
framework with a high density of sulfonic acid sites for proton conduction. Nat. Energy.

[68] Sharma A., Lim J., Jeong S., Won S., Seong J., Lee S., Kim Y. S., Baek S. B., Lah M. S. (2021). Superprotonic conductivity
of MOF-808 achieved by controlling the binding mode of grafted sulfamate. Angew. Chem., Int. Ed..

[69] Yang Y., He X., Zhang P., Andaloussi Y. H., Zhang H., Jiang Z., Chen Y., Ma S., Cheng P., Zhang Z. (2020). Combined intrinsic
and extrinsic proton conduction in robust covalent organic frameworks
for hydrogen fuel cell applications. Angew.
Chem., Int. Ed..

[70] Pal S. C., Mukherjee D., Sahoo R., Mondal S., Das M. C. (2021). Proton-conducting
hydrogen-bonded organic frameworks. ACS Energy
Lett..

[71] Ogasawara Y., Uchida S., Mizuno N. (2007). States of water in
ionic crystals
of [Cr_3_O­(OOCH)_6_(H2O)_3_]^+^ macrocation with α-Keggin-type polyoxometalates. Phys. Chem. C.

[72] Iwano T., Akutsu D., Ubukata H., Ogiwara N., Kikukawa Y., Wang S., Yan L., Kageyama H., Uchida S. (2024). Tuning proton
conduction by staggered arrays of polar Preyssler-type oxoclusters. J. Am. Chem. Soc..

[73] Iwano T., Shitamatsu K., Ogiwara N., Okuno M., Kikukawa Y., Ikemoto S., Shirai S., Muratsugu S., Waddell P. G., Errington R. J. (2021). Ultrahigh
proton conduction via extended
hydrogen-bonding network in a preyssler-type polyoxometalate-based
framework functionalized with a lanthanide ion. ACS Appl. Mater. Interfaces.

