# Supplementary material for: Dimensional Evolution from a Giant Molybdenum-Red Cage-like {Mo200} to 1D Chains Enabling Ultrahigh Proton Conduction
Source: J Am Chem Soc. 2026 Jun 23;148(26):27603–12. doi: 10.1021/jacs.6c06499 (PMC13352624; doi:10.1021/jacs.6c06499)
Supplement: Supplementary file 1 [file ja6c06499_si_001.pdf]

Supporting information

Dimensional Evolution from a Giant Molybdenum-Red Cage-like {Mo_{200}} to 1D Chains Enabling Ultrahigh Proton Conduction

Duidui Zhang‡,[a,c] Rongqing Tang‡,[a] Yubin Ma,[a] Qixin Zhao,[a] Qi Zheng,[b] Yongge Wei,*[c,d] De-Liang Long,*[e] Leroy Cronin*[e] and Weimin Xuan*[a]

[a] State Key Laboratory of Advanced Fiber Materials & College of Chemistry and Chemical Engineering, Donghua University, Shanghai 201620, China.

[b] State Key Laboratory of Advanced Fiber Materials & College of Materials Science and Engineering, Donghua University, Shanghai 201620, China.

[c] Department of Chemistry, Key Lab of Organic Optoelectronics and Molecular Engineering of Ministry of Education, Tsinghua University, Beijing 100084, China.

[d] Laboratory of Flexible Electronics Technology, Tsinghua University, Beijing 100084, China.

[e] School of Chemistry, The University of Glasgow, Glasgow G12 8QQ, United Kingdom.

‡ These authors contributed equally.

Table of contents

1. **Materials**
2. **Instrumentations**
3. **Synthetic procedures for compounds 1 and 2**
4. **Transmission electron microscopy (TEM) imaging**
5. **Single-crystal X-ray diffraction analyses**
6. **Redox titration**
7. **Bond valence sum (BVS) analysis**
8. **Structural analysis of 1 and 2**
9. **Crystallographic data and crystal structures of 1 and 2**
10. **Spectral characterization of 1 and 2**
11. **Proton conduction test of 1 and 2**
12. **References**

1. Materials

All chemicals were purchased commercially and used without further purification.

2. Instrumentations

Crystallography: Single crystal X-ray diffraction data of **1** was recorded on a Bruker/ARINAX MD2 diffractometer equipped with a MarCCD-300 detector at beam line station BL17B of Shanghai Synchrotron Radiation Facility (SSRF) at 150 K. Single crystal X-ray diffraction data of **2** was recorded on a Bruker D8 Venture instrument with Ga K_α radiation (λ = 1.34138).

Fourier-transform infrared (FT-IR) spectra: The IR was performed on a Nicolet iS50 FT-IR Spectrometer in the range of 500-4000 cm^{-1}. Intensities are denoted as w = weak, m = medium, s = strong, vs = very strong.

Powder X-ray diffraction (PXRD) measurements: The PXRD were collected on a Bruker D8 ADVANCE instrument with Cu K_α radiation (λ = 1.54056 Å) radiation in the range of $3° \leq 2\theta \leq 50°$ with a scanning rate of 0.02° s^{-1}.

Thermogravimetric Analysis (TGA): Thermogravimetric analysis was performed on a METTLER TOLEDO TG8000 Thermogravimetric Analyzer under nitrogen flow at a typical heating rate of 10 °C·min^{-1} from 30-800 °C.

Element Analyses: Element analyses for P, Mo, K and Na were performed on a Leeman Prodigy Plus inductivity-coupled plasma Inductively Coupled Plasma Optical Emission Spectroscopy (ICP-OES) and C, N and H content were determined by VARIDEL III elemental Analyzer.

Inductively Coupled Plasma Optical Emission Spectroscopy (ICP-OES): Elemental analyses for P, Mo, Na and K were determined with a Leeman Prodigy Plus inductivity-coupled plasma Inductively Coupled Plasma Optical Emission Spectroscopy (ICP-OES).

UV-Vis spectroscopy: The UV-Vis was performed on a UV3600 UV-Vis spectrophotometer.

Scanning electron microscope (SEM) analysis: The SEM was conducted on TESCAN MAIA 3, Czech Republic.

Transmission Electron Microscope (TEM): The TEM images and dark field images were collected by a JEM-2100F at an accelerating voltage of 200 kV.

Raman spectra: The Raman were recorded on an in Via Reflex laser Raman spectroscopy.

Electrospray Ionization-Mass Spectrometry (ESI-MS): The spectra were collected on a

Waters SYNAPT G2-Si mass spectrometer. Each sample solution was prepared by dissolving ca. 0.1 mg of sample in 1 mL of acetonitrile.

Proton conductivity: The dried single crystals were ground uniformly into powder. For each test, 55 mg of the powder sample was put into a homemade mold with a radius of 2.50 mm. Pellets were obtained via compression, and their exact thicknesses were measured using a vernier caliper. Silver glue was spread on both sides of the pellets and allowed to dry in the air. The pellet sample was attached with two copper electrodes. AC impedance measurements were performed on an electrochemical workstation CHI 760 at a frequency range from 1 MHz to 0.1 Hz. The specific relative humidity (RH) environments were controlled using different constant-humidity bottles, while the testing temperatures were finely regulated by a thermostatic water bath. Before each measurement, the sample pellet was allowed to stabilize at the target temperature and humidity for 1.5 hours to ensure complete equilibration.

Water adsorption: Water vapor adsorption isotherms were measured at 298.15 K on a BSD-660M A6B6M AdsorptoMeter (Beishide Instruments).

In situ infrared: In situ IR spectra of both compounds under water vapor were measured on a Nicolet iS50 Fourier Transform Infrared Spectrometer. Each powder sample was placed at the center of an IR cell and treated in vacuo at 423 K for 6 h. Then, the powder sample was exposed to water vapor at room temperature with equilibrium pressures of 0.1 MPa, 53%RH-98%RH. The equilibrium of the sorption was confirmed by the fact that the spectrum did not change over time. The spectra were deconvoluted with the Gaussian function.

3. Synthetic procedures for compounds 1 and 2

Compound 1: $Na_4(NH_4)_{42}H_{22}[(Mo^{VI}{}_8O_{26})_{0.5}Mo^{V}{}_{140}Mo^{VI}{}_{60}(OH)_{58}O_{524}(C_6H_5PO_3)_{10}]\cdot 250\ H_2O$, $Na_4(NH_4)_{42}H_{22}$\{**1a**\}$\cdot 250\ H_2O$

$(NH_4)_6Mo_7O_{24}\cdot 4H_2O$ (274 mg, 0.2 mmol) and $NH_2NH_2\cdot 2HCl$ (70 mg, 0.7 mmol) were dissolved in 6 mL of deionized water. The resulting mixture was stirred for about 15 min. After that, phenylphosphonic acid (79 mg, 0.5 mmol) was added. Then the mixture was stirred for another 0.5 h. Monitoring the pH, which should be around 2.4 with addition of 6 M HCl(aq). Afterwards, the mixture was transferred to a 10 mL vial with a metallic lid with a rubber septum and each vial was added with 20 μL 1M NaCl (aq), which was then heated at 120°C in an oven 3 days without any stirring. Dark red rhombus crystals suitable for X-ray crystallography were collected and washed with EtOH (yield 20 % based on Mo). IR (cm^{-1}): 3187 (s), 1421 (s), 1137 (w), 1097 (w), 956 (s), 883 (m), 667 (s), 548 (w). Raman spectrum (cm^{-1}): 989 (s), 816 (s), 659 (w), 372 (w), 335 (w), 280 (m). Elemental analysis % calcd (found): H 2.27 (2.11), N 1.62

(1.80), C 1.99 (1.93), P 0.85 (0.83), Na 0.25 (0.29), Mo 53.97 (54.52).

Compound 2: $K_4(NH_4)_{44}H_{24}[(Mo^{VI}{}_8O_{26})_{0.5}Mo^{V}{}_{140}Mo^{VI}{}_{66}(OH)_{58}O_{544}(C_6H_5PO_3)_{10}]\cdot 260\ H_2O$, $K_4(NH_4)_{44}H_{24}\{$**2a**$\}\cdot 260\ H_2O$

$(NH_4)_6Mo_7O_{24}\cdot 4H_2O$ (432 mg, 0.35 mmol) and $NH_2NH_2\cdot 2HCl$ (50 mg, 0.5 mmol) were dissolved in 6 mL of deionized water. The resulting mixture was stirred for about 15 min. After that, phenylphosphonic acid (79 mg, 0.5 mmol) was added. Then the mixture was stirred for another 0.5 h. Monitoring the pH, which should be around 2.4 with addition of 6 M HCl(aq). Afterwards, the mixture was transferred to a 10 mL vial with a metallic lid with a rubber septum and each vial was added with 50 µL 1M KCl (aq), which was then heated at 120°C in an oven 3 days without any stirring. Dark red rhombus crystals suitable for X-ray crystallography were collected and washed with EtOH (yield 14 % based on Mo). IR (cm^{-1}): 3184 (s), 1419 (s), 1102 (w), 1061 (w), 954 (s), 879 (m), 658 (s), 553 (w). Raman spectrum (cm^{-1}): 990 (s), 817 (s), 661 (w), 372 (w), 335 (w), 281 (m). Elemental analysis % calcd (found): H 2.20 (2.25), N 1.67 (1.63), C 1.96 (1.91), P 0.84 (0.87), K 0.42 (0.39), Mo 54.28 (54.75).

4. Transmission electron microscopy (TEM) imaging

Due to the easy aggregation of **1** during the TEM imaging when preparing the sample by evaporation of the aqueous solution**,** cation exchange was performed by adding excessive tetrabutylammonium bromide (TBA·Br) into the aqueous solution of **1** to afford TBA-**1**, which is highly soluble in acetonitrile and shows good monodispersity for TEM imaging. The detailed process can be described as follow (Scheme S1): **1** (5 mg) was dispersed in 5 mL deionized water with stirring overnight to form brown aqueous solution. Afterwards, this solution was added to an aqueous solution of tetrabutylammonium bromide (300 mg in 5 mL water), and brown precipitation was formed immediately. The precipitate was collected by centrifugation, and washed with H_2O and EtOH. Then the as-synthesized TBA-**1** was dissolved in acetonitrile to prepare monodispersed clusters on a copper mesh for TEM imaging. The TEM imaging was performed on JEM-2100F transmission electron microscopy.

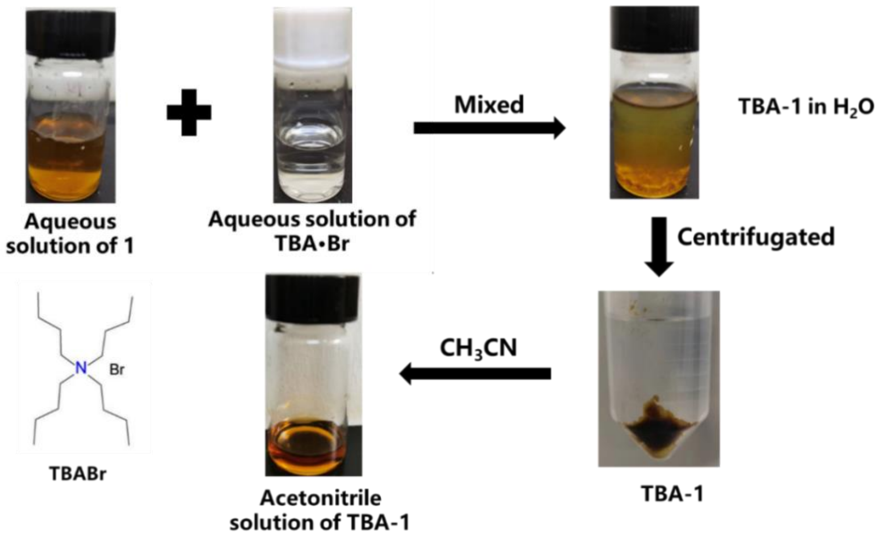

Scheme S1. The procedure for preparation of TBA-**1**.

5. Single-crystal X-ray diffraction analyses

Suitable single crystals were selected and mounted onto a rubber loop using Fomblin oil. Data collection and reduction were performed using the Apex 4 software package. Structure solution and refinement were carried out by SHELXS-2014[1] and SHELXL-2018[2] using Olex2.[3] Corrections for incident and diffracted beam absorption effects were applied using empirical absorption correction. The disordered {Mo_8} template in compound **1** and **2** was modelled and refined isotropically with half occupancy. Some of the O atoms on {Mo_4} building blocks show low Ueq and are subjected to isotropic refinement as well. Solvent water molecules with reasonable Ueq are located from residual electron density map, while the relatively weak Q peaks are squeezed by applying 'Solvent mask'[4] embedded in Olex-2.[3] Crystallographic formulas typically contain many more water molecules in the crystal lattice than found in the sample after drying. Overall, the final refinement statistics are pretty good, and in all cases the structural analysis allows us to unambiguously determine the structures of the compounds. In compound **2**, the linker {Mo_8} unit was refined with 88% occupancy and twofold disorder at the Mo55 and Mo56 sites, reflecting two orientations of this {Mo_8} unit. The remaining 12% occupancy in this region is attributed to a residual M54 fragment, carried over from compound **1**. For simplicity, the reported formula rounds the linker {Mo_8} unit up to full occupancy. All the structures of compounds **1** and **2** were deposited at Cambridge Crystallographic Data Center, the data can be obtained *via* www.ccdc.cam.ac.uk/data_request/cif under deposition numbers of 2321973 and 2538217.

6. Redox titration

The cerimetric titration was carried out using a 0.005 M solution of Ce^{IV} in 0.5 M of sulphuric acid as oxidant which was added dropwise to a solution of **1** (5 mg in 15 mL of H_2O). After addition of 4.00 mL of the oxidant the color of the solution turned from light orange red to colorless along with a characteristic potential jump showed the presence of 140 ± 5 d electrons, which (formally) corresponds to 140 Mo^V centers (theoretical value for 140 e^- reduced species: 3.86 mL). Due to the poor solubility of compound **2**, redox titration was performed exclusively on compound **1** to determine the number of reduced electrons.

7. Bond valence sum (BVS) analysis

Bond valence sum calculations were performed for each bond using the bond distance (R) measured and empirical parameters R_0 and B: bond valence = EXP((R_0-R)/B). BVS for each metal center was then summed from all bond valences of the bonds listed. The parameters R_0 and B were taken from Gagne & Hawthorne.[5] Mo(6+), Mo(5+) and P(5+) centers were calculated separately using corresponding parameters listed below. Parameter list:

Bond	R_0	B
MO(6+)-O	1.903	0.349
MO(5+)-O	1.888	0.314
P(5+)-O	1.615	0.37
P(5+)-C	1.890	0.37

Table S1. BVS values for Mo and P atoms in **1a**.

Atom	BVS	Atom	BVS	Atom	BVS	Atom	BVS
Mo1	5.637	Mo17	5.948	Mo33	4.943	Mo49	4.782
Mo2	5.126	Mo18	4.631	Mo34	4.783	Mo50	6.033
Mo3	4.958	Mo19	6.109	Mo35	4.838	Mo51	5.015
Mo4	5.065	Mo20	6.149	Mo36	4.907	Mo52	4.903
Mo5	4.960	Mo21	4.767	Mo37	4.842	Mo53	4.917
Mo6	4.914	Mo22	6.016	Mo38	4.971	Mo54	5.847
Mo7	6.144	Mo23	4.842	Mo39	5.777	P1	5.088
Mo8	6.007	Mo24	4.981	Mo40	5.734	P2	5.073
Mo9	4.809	Mo25	4.875	Mo41	6.076	P3	4.891
Mo10	4.976	Mo26	6.097	Mo42	4.745		
Mo11	4.963	Mo27	5.033	Mo43	4.775		
Mo12	4.995	Mo28	4.936	Mo44	5.304		
Mo13	4.867	Mo29	4.969	Mo45	4.701		
Mo14	6.022	Mo30	4.977	Mo46	6.082		
Mo15	4.861	Mo31	6.029	Mo47	5.014		
Mo16	4.917	Mo32	6.023	Mo48	4.885		

[a] the Mo atoms on central disordered *β*-{Mo_8} are not subjected to BVS owing to the high disorder

Table S2. BVS values for Mo and P atoms in **2a**.

Atom	BVS	Atom	BVS	Atom	BVS	Atom	BVS
Mo1	5.896	Mo17	4.943	Mo33	5.960	Mo49	4.869
Mo2	5.793	Mo18	4.986	Mo34	5.030	Mo50	4.971
Mo3	5.985	Mo19	4.880	Mo35	4.905	Mo51	6.051
Mo4	4.900	Mo20	4.789	Mo36	4.735	Mo52	4.924
Mo5	4.948	Mo21	4.993	Mo37	5.026	Mo53	4.910
Mo6	4.967	Mo22	6.025	Mo38	5.991	Mo54	5.345
Mo7	4.958	Mo23	4.985	Mo39	6.001	P1	5.081
Mo8	5.938	Mo24	5.980	Mo40	4.781	P2	5.006
Mo9	6.015	Mo25	4.874	Mo41	5.041	P3	5.036
Mo10	4.868	Mo26	6.060	Mo42	6.014		
Mo11	4.966	Mo27	4.943	Mo43	4.817		
Mo12	4.929	Mo28	6.000	Mo44	4.987		
Mo13	4.938	Mo29	4.843	Mo45	6.085		
Mo14	6.126	Mo30	4.931	Mo46	4.871		
Mo15	4.924	Mo31	4.713	Mo47	4.763		
Mo16	4.941	Mo32	4.985	Mo48	4.947		

Table S3. BVS values for O atoms of hydroxyl and coordination waters in **1** and **2**.

Compounds	Atom	BVS	Atom	BVS	Atom	BVS
**1**	O12	1.222	O23	1.103	O26	1.047
	O53	1.094	O56	1.108	O59	1.206
	O78	1.809	O82	1.020	O94	1.093
	O102	1.208	O109	1.124	O117	1.090
	O119	1.095	O137	1.191	O147	1.129
	O154	1.071				
**2**	O9	1.057	O15	1.218	O16	1.079
	O17	1.061	O22	1.174	O26	1.184
	O45	1.073	O78	1.141	O80	1.197
	O87	1.119	O88	1.095	O100	1.234
	O103	1.101	O117	1.064	O120	1.184
	O128	1.101	O143	1.090		

Table S4. The numbers and coordinated modes of the Mo and P atoms in **1** and **2**.

**Complex**	**1**	**2**
**Mo**	204 Mo total = 64 Mo^{VI} + 140 Mo^{V}<br>18 four coordinated Mo centers and<br>186 six-coordinated Mo centers	210 Mo total = 70 Mo^{VI} + 140 Mo^{V}<br>18 four-coordinated Mo centers and<br>192 six-coordinated Mo centers
**P**	10 P total	10 P total

8. Structural analysis of 1 and 2

The structural analysis and formula determination of the mix-valence Mo clusters has been well established in literatures, and it in general requires a variety of analytical techniques, including redox titration (to determine the number of reduced Mo atoms), bond valence sum analysis (BVS), X-ray photoelectron spectrum (XPS), elemental analysis and thermogravimetric analysis (TGA) along with single-crystal X-ray diffraction analysis. Herein, **1** was selected to exemplify the general approach used to determine the formula, and the compound **2** is treated in the same way.

Compound 1: Firstly, the overall reduction state of **1** (140 electrons reduced) was confirmed using three independent techniques (XPS, redox titration and bond valence sum analysis). The Mo 3d XPS spectrum of **1** suggests both Mo^{VI} and Mo^{V} species are presented in **1**. BVS calculations were carried out on all the Mo and O centers, revealing that **1** is composed of a 140-electron reduced anionic skeleton containing 58 singly protonated oxygen atoms which are located at the positions of μ_3-O on {Mo_4}, μ_2-O between two dumbbell-shaped {Mo_2} units in {Mo_5L} and tripodal {Mo_6} as well as μ_2-O between two {Mo_5L} units. Moreover, all the other bridging O atoms and terminal O atoms are considered as O^{2-} based on BVS. However, BVS could not be applied to β-{Mo_8} due to the high disorder of these groups. Hence, all the Mo atoms on central disordered β-{Mo_8} are assumed to be Mo^{VI}, consistent with the reported β-{Mo_8}.[6] In addition, the redox titration experiment reveals that the overall reduction state of **1** is 140 ± 5 e^-, matching quite well with theoretical 140 Mo^{V} centers. Therefore, the oxidation states of Mo should be +5 for 140 Mo atoms and +6 for 64 Mo atoms in **1**. Coupled with the molecular structure determined by single-crystal X-ray diffraction data, the formula and overall charge could be assigned for **1a** as $[(Mo^{VI}_8O_{26})_{0.5}Mo^{V}_{140}Mo^{VI}_{60}(OH)_{58}O_{524}(C_6H_5PO_3)_{10}]^{68-}$. To balance the negative charge of -68, 4 sodium ions, 42 ammonium cation and 22 protons are proposed as counterions based on elemental analysis results of Na, N and H. The amount of

phenylphosphonate is deduced from C, P and H analysis, and a total number of 10 phenylphosphonates are found in the structure of **1**. Finally, TGA curve of **1** exhibits a total weight loss of 12.1 % from r.t. to 150 °C, which corresponds to ~250 guest water molecules. Taking into consideration of the obtained information from the above calculations and data analysis, the formula of **1** could therefore be determined as $Na_4(NH_4)_{42}H_{22}[(Mo^{VI}{}_8O_{26})_{0.5}Mo^{V}{}_{140}Mo^{VI}{}_{60}(OH)_{58}O_{524}(C_6H_5PO_3)_{10}]\cdot 250\ H_2O$

$\equiv Na_4(NH_4)_{42}H_{22}\{$**1a**$\}\cdot 250\ H_2O$

Compound 2: Adopting the same procedure as **1**, the formula of **2** was determined as $K_4(NH_4)_{44}H_{24}[(Mo^{VI}{}_8O_{26})_{0.5}Mo^{V}{}_{140}Mo^{VI}{}_{66}(OH)_{58}O_{544}(C_6H_5PO_3)_{10}]\cdot 260\ H_2O$

$\equiv K_4(NH_4)_{44}H_{24}\{$**2a**$\}\cdot 260\ H_2O$

9. Crystallographic data and crystal structures of 1 and 2

Table S5. Crystallographic Details for Compound 1 and 2

Complex	1	2
Empirical formula	$Mo_{204}O_{885}C_{60}P_{10}Na_4N_{42}H_{818}$	$Mo_{210}O_{905}C_{60}P_{10}K_4N_{44}H_{828}$
Formula weight	36266.3457	37264.47
Temperature (K)	150.00	150.00
Crystal system	Orthorhombic	Orthorhombic
Space group	*Cccm*	*Cccm*
a/Å	42.351(2) Å	42.100(2) Å
b/Å	45.283(5) Å	45.235(3) Å
c/Å	58.626(3) Å	58.723(3) Å
α(°)	90	90
β(°)	90	90
γ(°)	90	90
V/Å^3	112434(15)	111831(11)
Z	4	16
D_c(g/cm^3)	1.959	2.036
F(000)	61592	63654
Reflections collected	837807	1506397
Independent reflections	50628 [R_{int} = 0.0584]	64647 [R_{int} = 0.0564]
GOF	1.153	1.042
Final R indices [I>2σ(I)]	R_1 = 0.0794, wR_2 = 0.1727	R_1 = 0.0467, wR_2 = 0.1293
R indices (all data)	R_1 = 0.0827, wR_2 = 0.1742	R_1 = 0.0485, wR_2 = 0.1306

$^aR_1 = \Sigma||F_o| - |F_c||/\Sigma|F_o|$, $^bwR_2 = [\Sigma w(F_o^2 - F_c^2)^2/\Sigma w(F_o^2)^2]^{1/2}$.

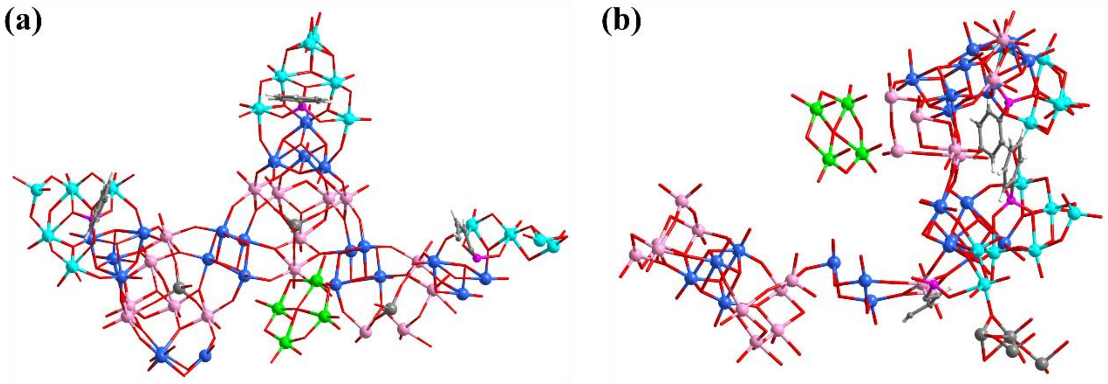

Figure S1. The asymmetric units of **1** (a) and **2** (b). Color code: cyan, blue, pink, gray and green, Mo; red, O; pink, P; gray, C; white, H.

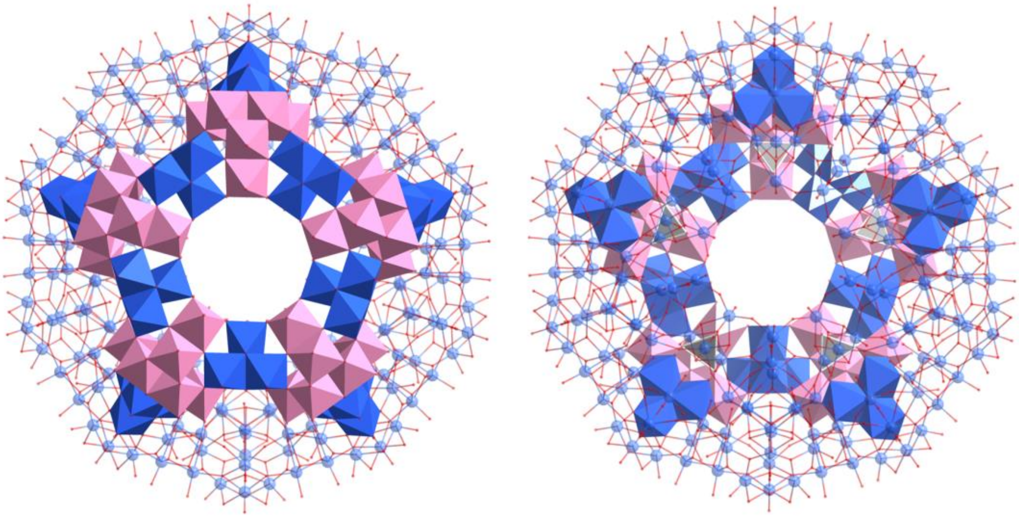

Figure S2. The $\{Mo_{75}\}$ structure fragments in $\{Mo_{260}\}$. Color code: pink and blue, Mo; red, O.

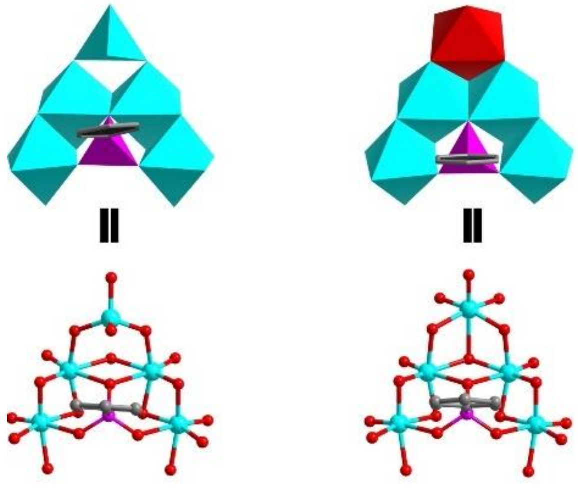

Figure S3. Polyhedron and Ball-and-stick representation of $\{Mo_5L\}$ and $\{Mo_5L\}^*$ BBs in **1**. Color code: cyan, Mo; red, O; pink, P; gray, C.

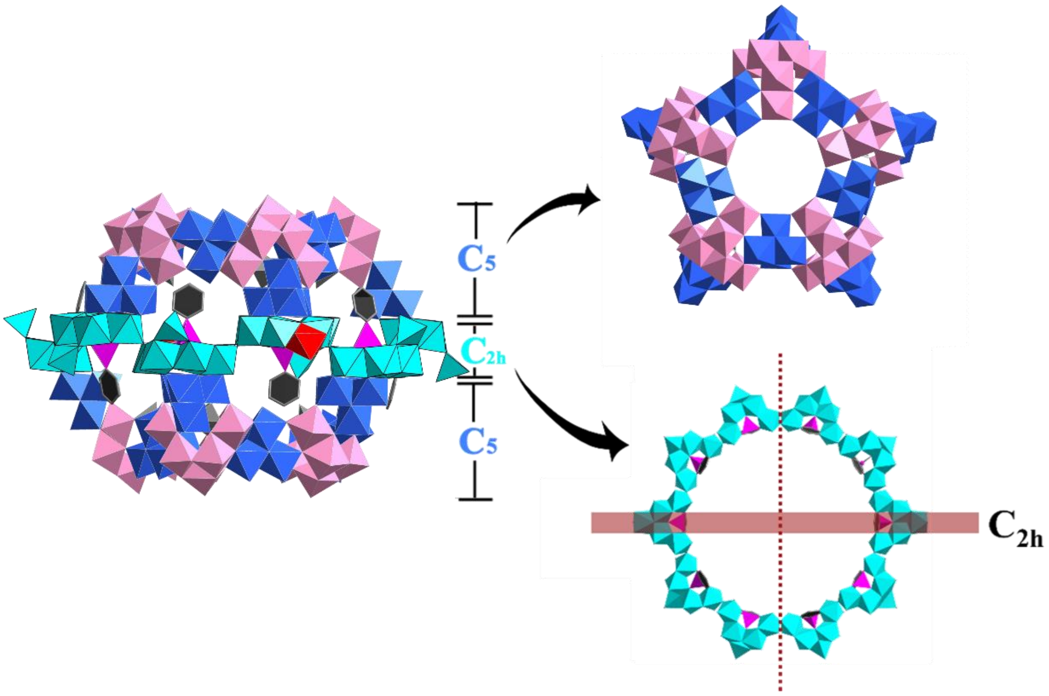

Figure S4. Top view of the symmetry breaking from C_5-symmetric {Mo_{75}} to C_{2h}-symmetric central belt consisted of eight {Mo_5L} and two {Mo_5L}* BBs. Color code: cyan, orange, blue and light pink, Mo; red, O; pink, P; gray, C.

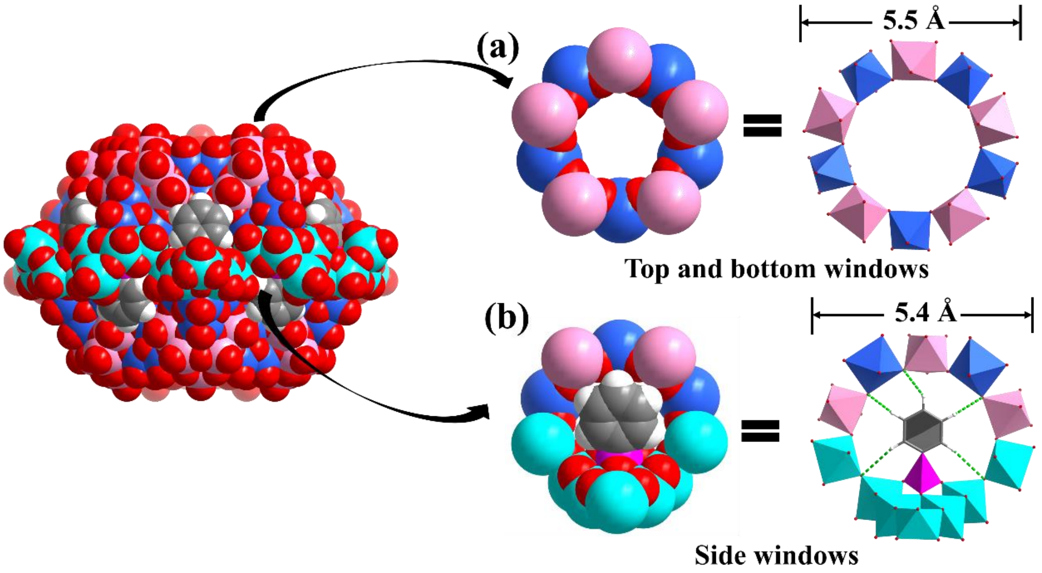

Figure S5. View of the pentagonal windows (a) and side windows (b) shown in space filling and polyhedral modes in **1**. The hydrogen bonding between phenyl ring of L and the bridging O atoms are highlighted in dotted green line.

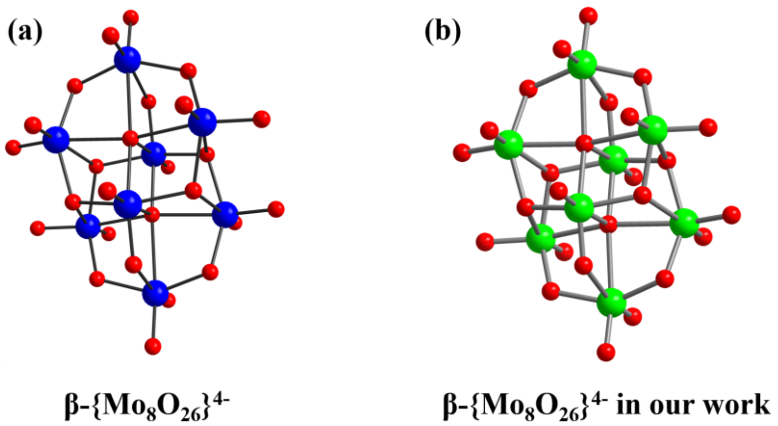

Figure S6. The ball-and-stick representation of *β*-{Mo_8} reported in literature[6,7] (a) and *β*-{Mo_8} observed in **1a** (b). Color code: blue and green, Mo; red, O.

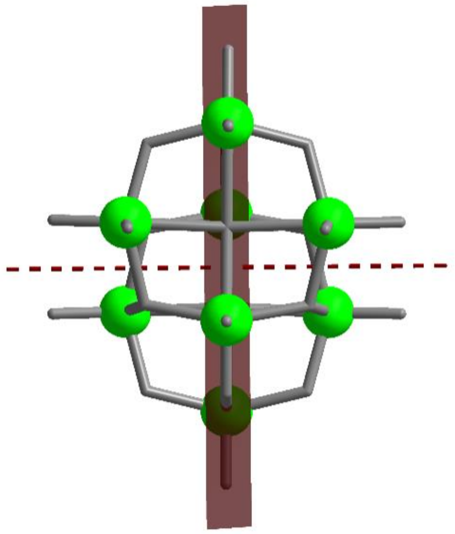

Figure S7. View of the C_{2h} symmetry of *β*-{Mo_8} in **1a**. The mirror plane going through *β*-{Mo_8} is shown by dark-red rectangle and the C_2 axis is represented by dark-red dotted line. Color code: green, Mo; gray, O.

10. Spectral characterization of 1 and 2

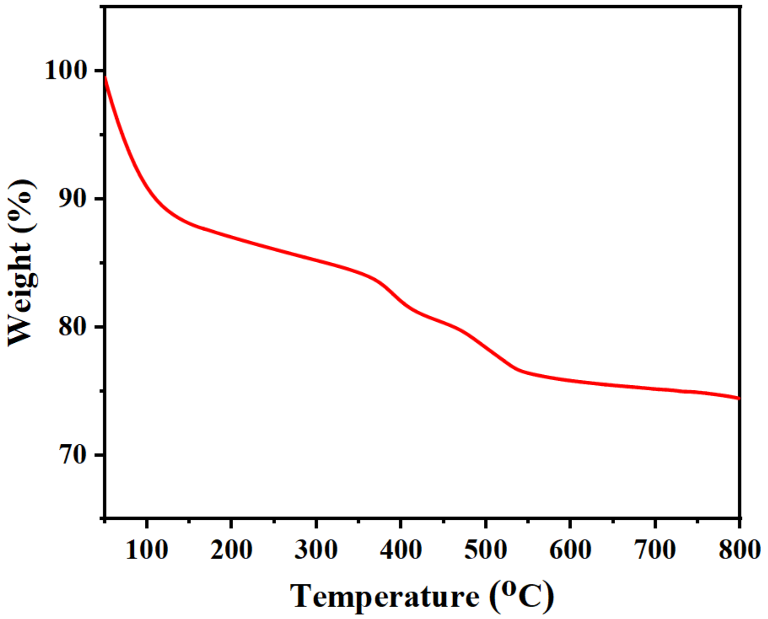

Figure S8. The thermogravimetric curve of **1**. The weight loss of 12.1% between r.t. and 150 °C corresponds to ~ 250 guest H_2O.

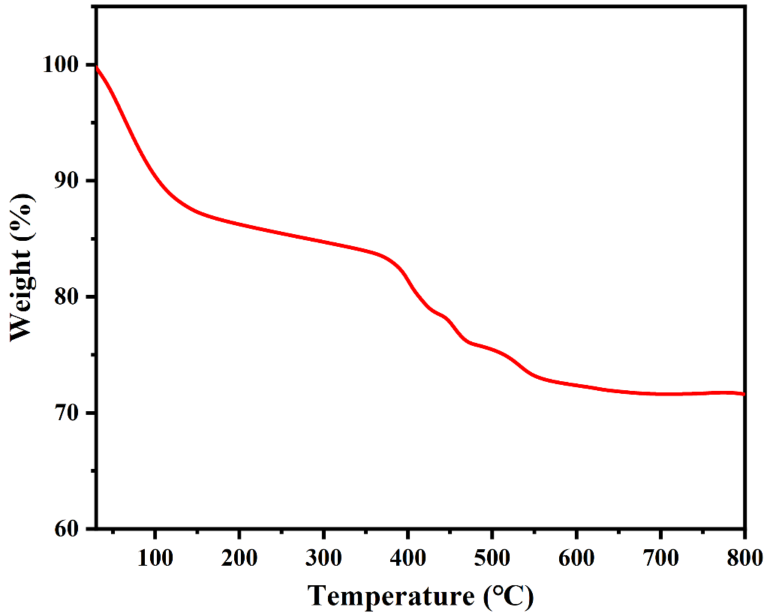

Figure S9. The thermogravimetric curve of **2**. The weight loss of 12.7% between r.t. and 150 °C corresponds to ~ 260 guest H_2O.

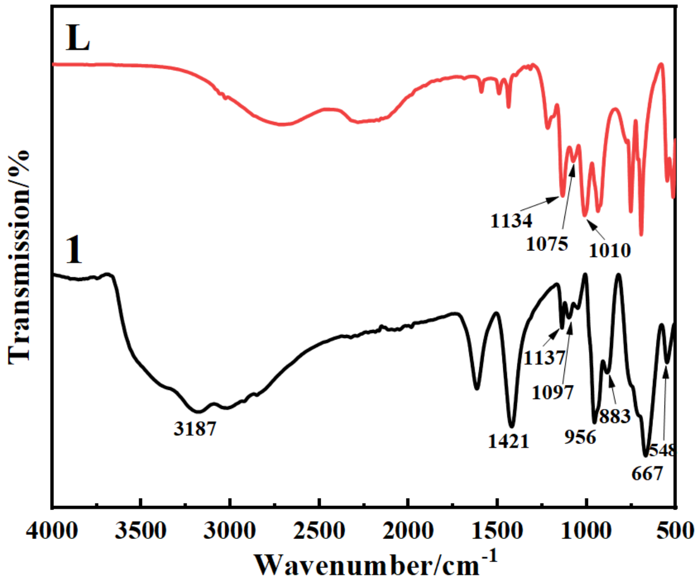

Figure S10. FT-IR spectra of **1** (black line) and **L** (red line).

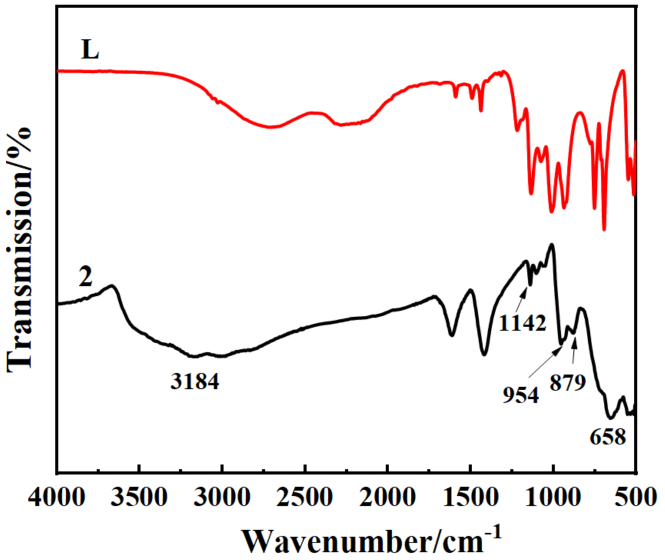

Figure S11. FT-IR spectra of **2** (black line) and **L** (red line).

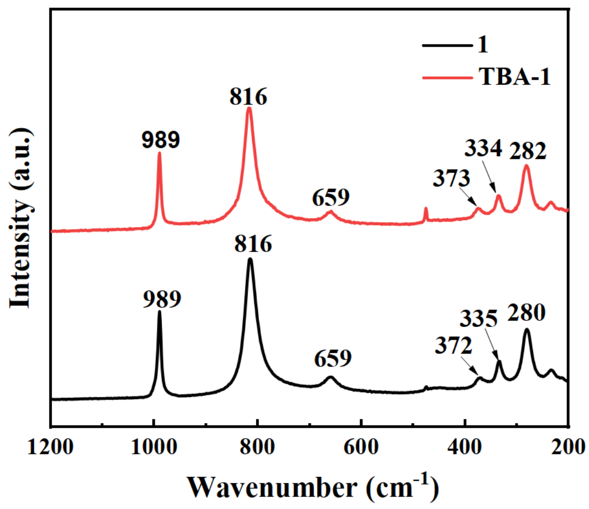

Figure S12. Raman spectra of **1** and TBA-**1**.

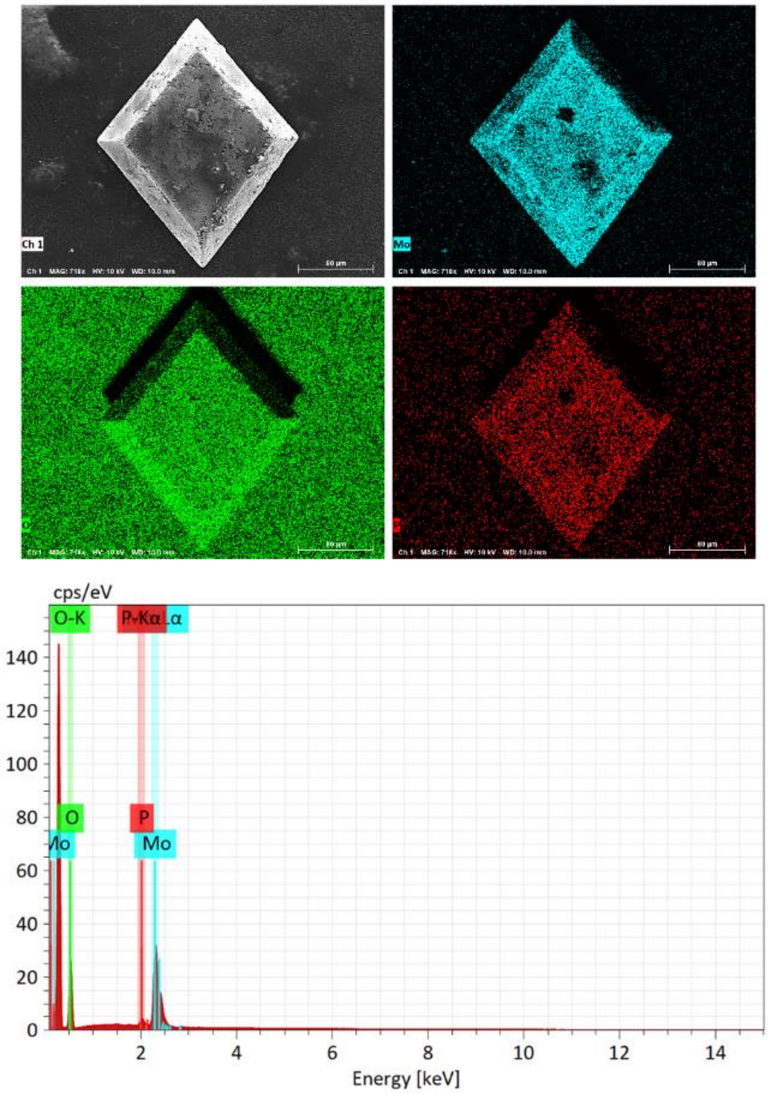

Figure S13. Elemental mapping images (Top) and EDS (bottom) spectrum of **1**.

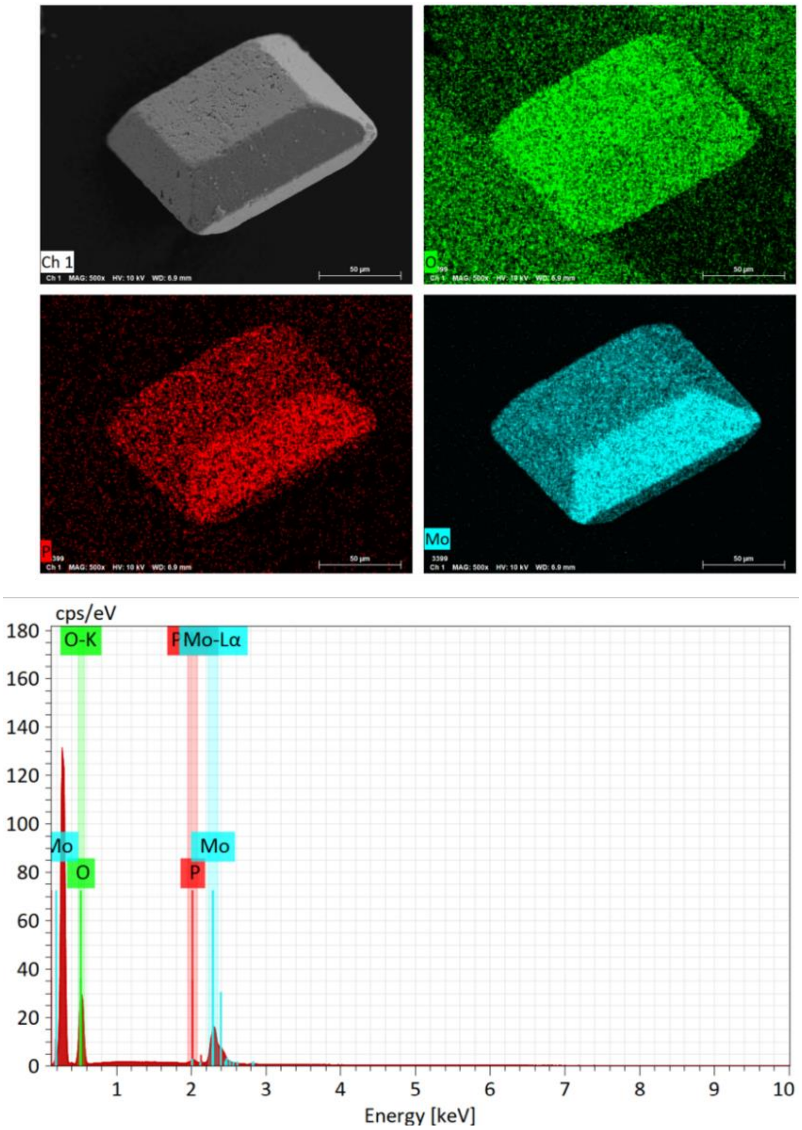

Figure S14. Elemental mapping images (Top) and EDS (bottom) spectrum of **2**.

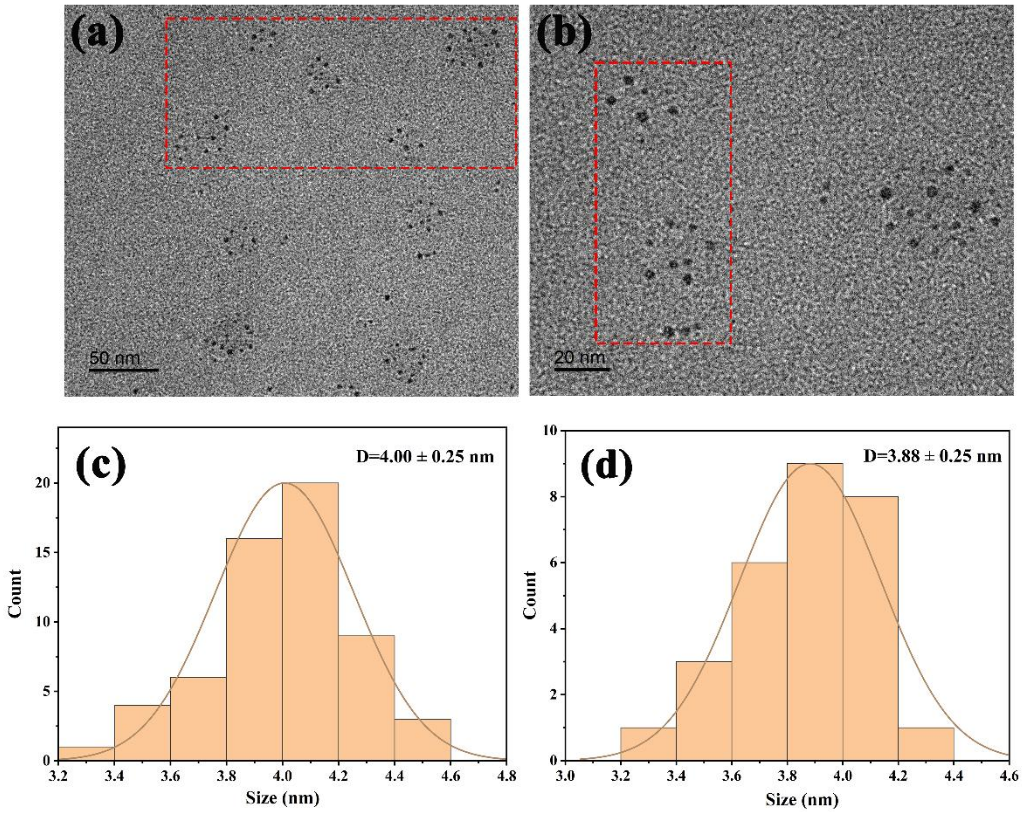

Figure S15. (a-b) TEM images of TBA-**1** and (c-d) Particle size distribution images of TBA-**1** at 50 nm and 20 nm scale bar, respectively. Images a and b show the good monodispersity of cage-shaped **1** with uniform size. Images c and d indicate the identifiable uniform dots with size of 4.00 ± 0.25 nm and 3.88 ± 0.25 nm, comparing well with the core size of 2.5 nm ×4.1 nm for **1**.

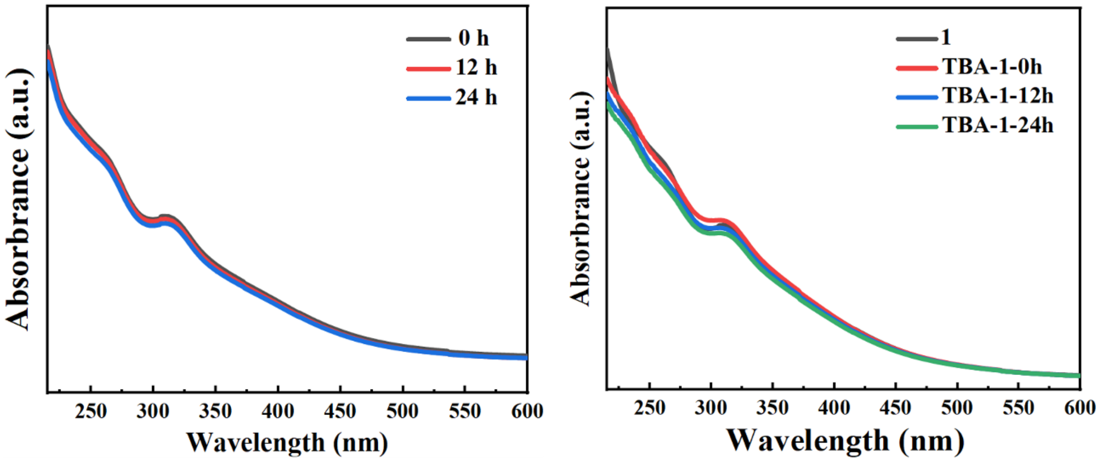

Figure S16. UV-Vis absorption spectra of **1** in aqueous solution for 24h (a) and UV−vis absorption spectra of TBA-**1** in acetonitrile for 24 h (b).

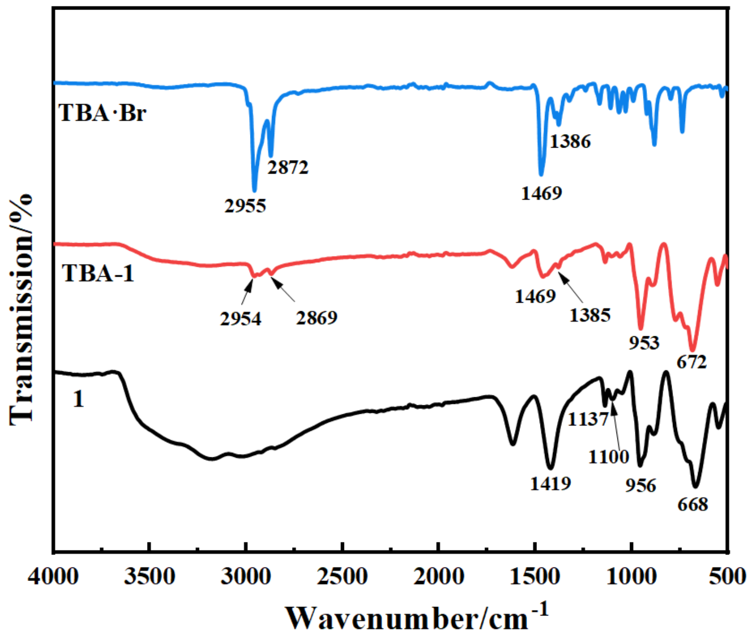

Figure S17. FT-IR spectra of TBA·Br, TBA-**1** and **1**. The characteristic bands of TBA·Br (2955, 2872, 1469 and 1386 cm^{-1}) could be seen clearly in the FT-IR spectrum of TBA-**1**, confirming the existence of TBA. For TBA-**1**, the two strong bands (around 953 and 672 cm^{-1}) attributed to the Mo=O and Mo-O-Mo vibrations can be related to the two peaks centered at 956 and 668 cm^{-1} in the spectrum of **1**.

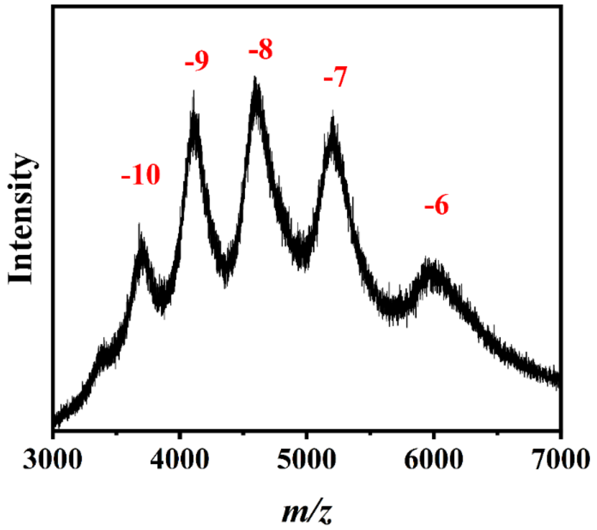

Figure S18. ESI-MS spectrum of TBA-**1** in a CH_3CN solution.

Table S6. Mass spectral analysis of TBA-**1** in CH_3CN, with proposed formula and charge of peaks

z	m/z (Obs.)	m/z (Cal.)	Assignment
-10	3655.35	3655.61	$[Mo_{204}(OH)_{58}O_{537}(C_6H_5PO_3)_{10}](C_{16}H_{36}N)_{19}H_{39}(CH_3CN)_{20}(H_2O)_{21}$
-9	4109.91	4109.86	$[Mo_{204}(OH)_{58}O_{537}(C_6H_5PO_3)_{10}](C_{16}H_{36}N)_{20}H_{39}(CH_3CN)_{22}(H_2O)_{27}$
-8	4606.58	4606.70	$[Mo_{204}(OH)_{58}O_{537}(C_6H_5PO_3)_{10}](C_{16}H_{36}N)_{20}H_{40}(CH_3CN)_{20}(H_2O)_{24}$
-7	5219.90	5219.46	$[Mo_{204}(OH)_{58}O_{537}(C_6H_5PO_3)_{10}](C_{16}H_{36}N)_{20}H_{41}(CH_3CN)_{14}(H_2O)_{20}$
-6	5955.31	5955.13	$[Mo_{204}(OH)_{58}O_{537}(C_6H_5PO_3)_{10}](C_{16}H_{36}N)_{17}H_{45}(CH_3CN)_{12}(H_2O)_{20}$

11. Proton conduction test of 1 and 2

Proton Conductivity Calculation

The proton conductivity of the pellet sample at specified temperatures (30 to 80 °C) and relative humidities (53% to 98% RH) was calculated utilizing the following equation:

$$\sigma = L / (R \times S)$$

where σ stands for the proton conductivity (S cm^{-1}), L is the measured thickness of the compressed pellet (cm), S corresponds to the cross-sectional area of the pellet (cm^2), and R is the bulk resistance of the sample (Ω) extracted from the semi-circle of the AC impedance Nyquist plot measured at the corresponding temperature and humidity. For the measurements in this work, the homemade mold has a radius of 2.50 mm, yielding a constant cross-sectional area (S) of 0.196 cm^2. Due to slight variations in the compression process and sample amounts, the actual measured thicknesses (L) of these pellets varied within a range of 1.20 ~ 1.25 mm. Importantly, for each specific measurement at a given condition, the exact thickness of that individual pellet was precisely recorded and strictly applied to the calculation of its respective conductivity.

Determination of Bulk Resistance

The bulk resistance (R) of the pellet samples, which was utilized for the conductivity calculation, was determined based on the characteristic profiles of the Nyquist plots under varying testing conditions.

Under conditions where a distinct semi-circle at higher frequencies and a polarization tail at lower frequencies were observed, the Nyquist plots were fitted with an equivalent circuit model. This model comprises a contact resistance (R1), the bulk resistance of the sample (R2), a constant phase element (CPE1) representing the non-ideal capacitance, and a Warburg diffusion element (W1).

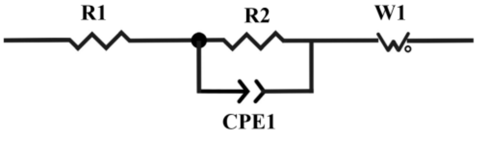

Conversely, at elevated temperatures or high relative humidities, the significantly enhanced proton conductivity caused the characteristic frequency of the bulk conduction process to exceed the upper frequency limit of the impedance analyzer (1 MHz). Consequently, the high-frequency semi-circle disappeared, leaving only an inclined low-frequency polarization tail. In such scenarios, the bulk resistance (R) was directly determined by reading the high-frequency intercept of the impedance curve with the real axis (Z').

Activation Energy Calculation

To investigate the proton conduction mechanism, the activation energy (E_a) was evaluated based on the standard Arrhenius equation:

$$\sigma T = \sigma_0 \exp(-E_a/k_B T)$$

where σ is the proton conductivity (S cm^{-1}), T is the absolute temperature (K), σ_0 is the pre-exponential factor (S cm^{-1} K), E_a is the activation energy for proton transfer (eV), and k_B is the Boltzmann constant. By taking the natural logarithm on both sides, the equation can be expressed as $\ln(\sigma T) = \ln(\sigma_0) - E_a/(k_B T)$. The activation energy (E_a) can thus be directly determined from the slope of the linear fit by plotting $\ln(\sigma T)$ versus 1000/T.

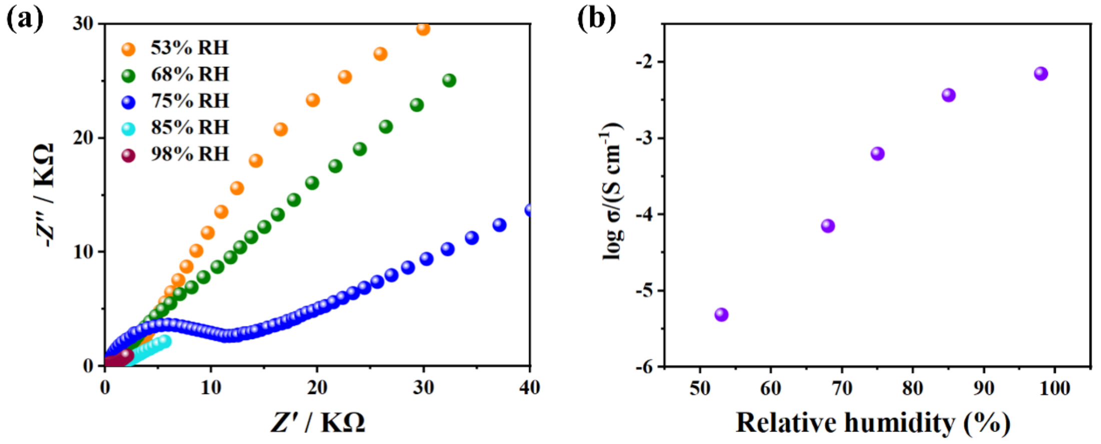

Figure S19. (a) Nyquist plots of compound **1** at 30 °C under various relative humidities (53%-98% RH). (b) Humidity-dependent proton conductivity of compound **1** at 30 °C. For clarity, the proton conductivity (σ) values are plotted on a logarithmic scale.

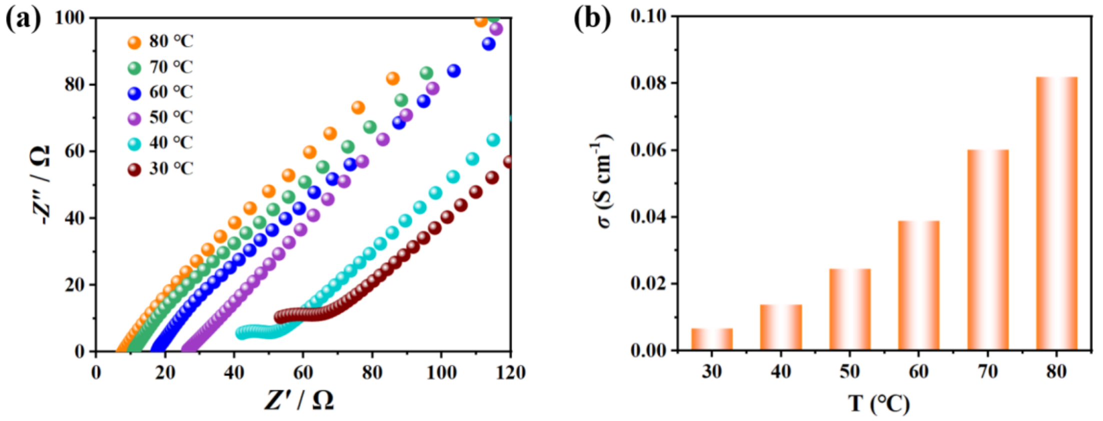

Figure S20. (a) Nyquist plots of compound **1** at 98% RH and various temperatures ranging from 30 to 80 °C. (b) Temperature-dependent proton conductivity of compound **1** at 98% RH.

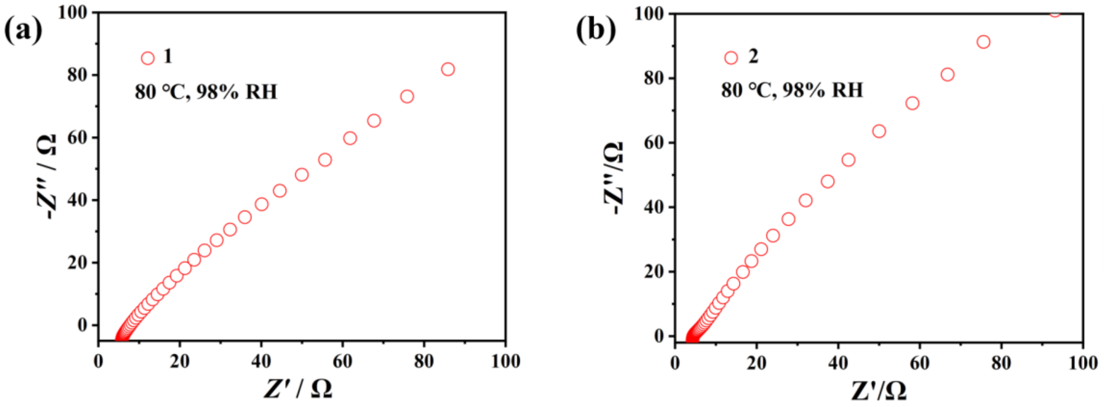

Figure S21. The Nyquist plots of compound **1** and **2** at 80°C and 98% RH.

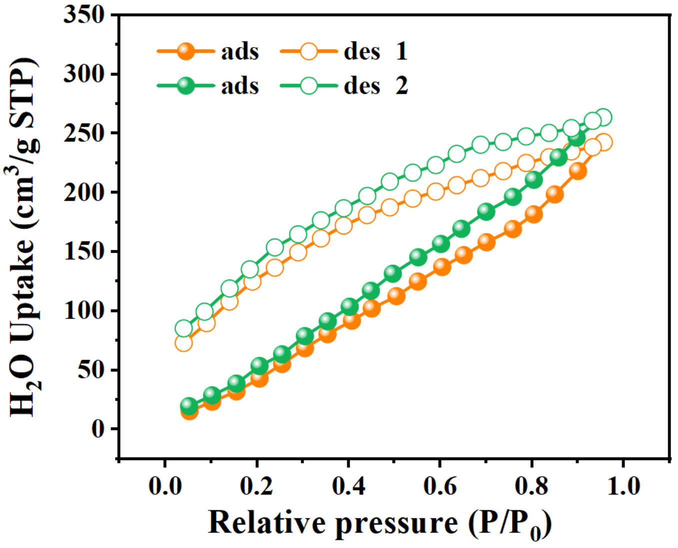

Figure S22. Water vapor sorption isotherms of **1** and **2**.

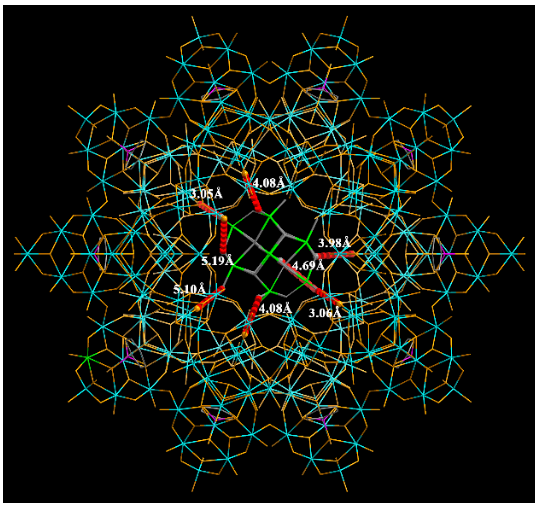

Figure S23. The hydrogen bonding network inside the cavity by the terminal oxygen atoms of the β-{Mo_8} template and framework along the b axis.

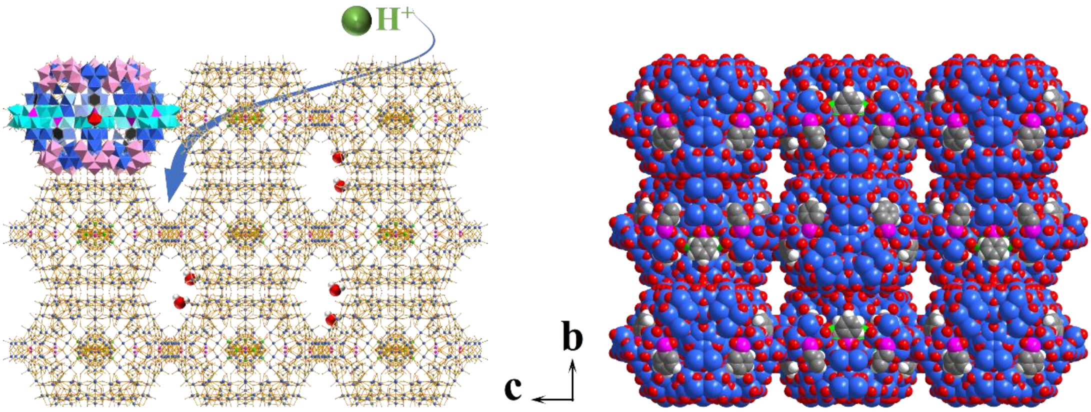

Figure S24. Packing model of **1** along a-axis.

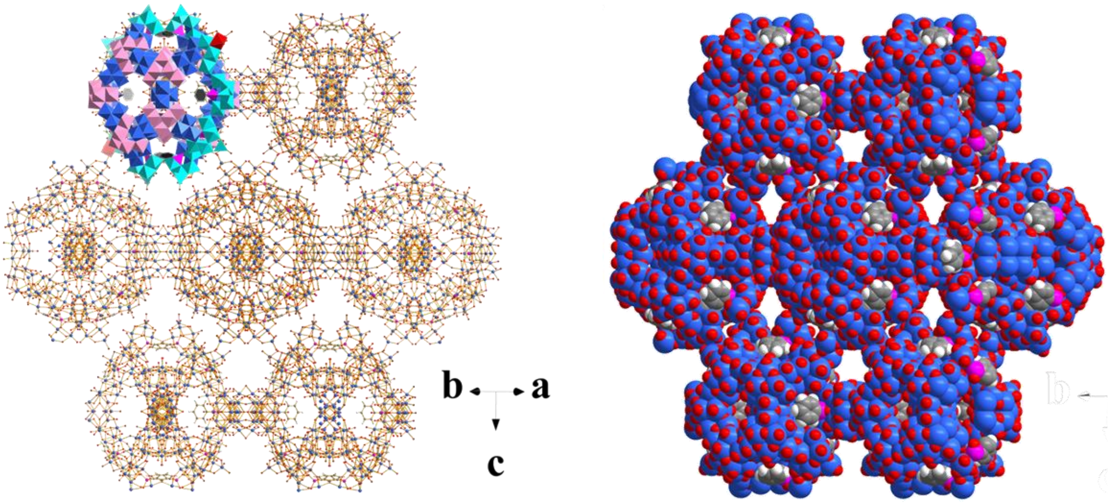

Figure S25. Packing model of **2** along above direction.

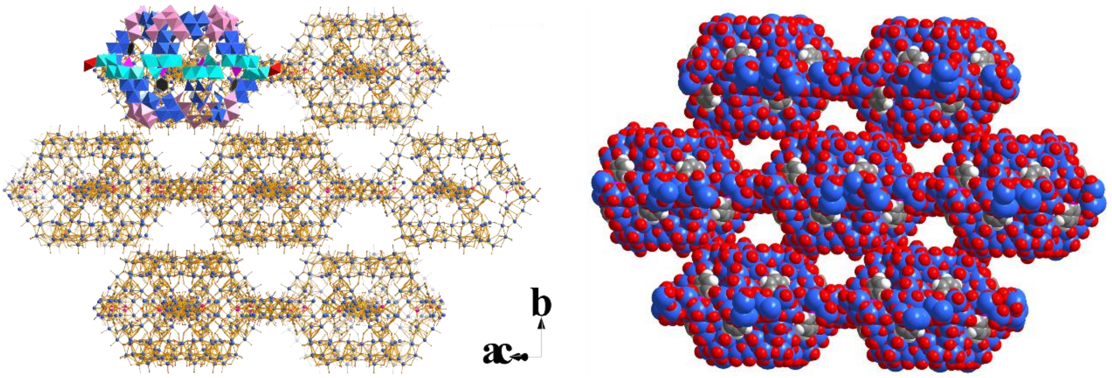

Figure S26. Packing model of **2** along above direction.

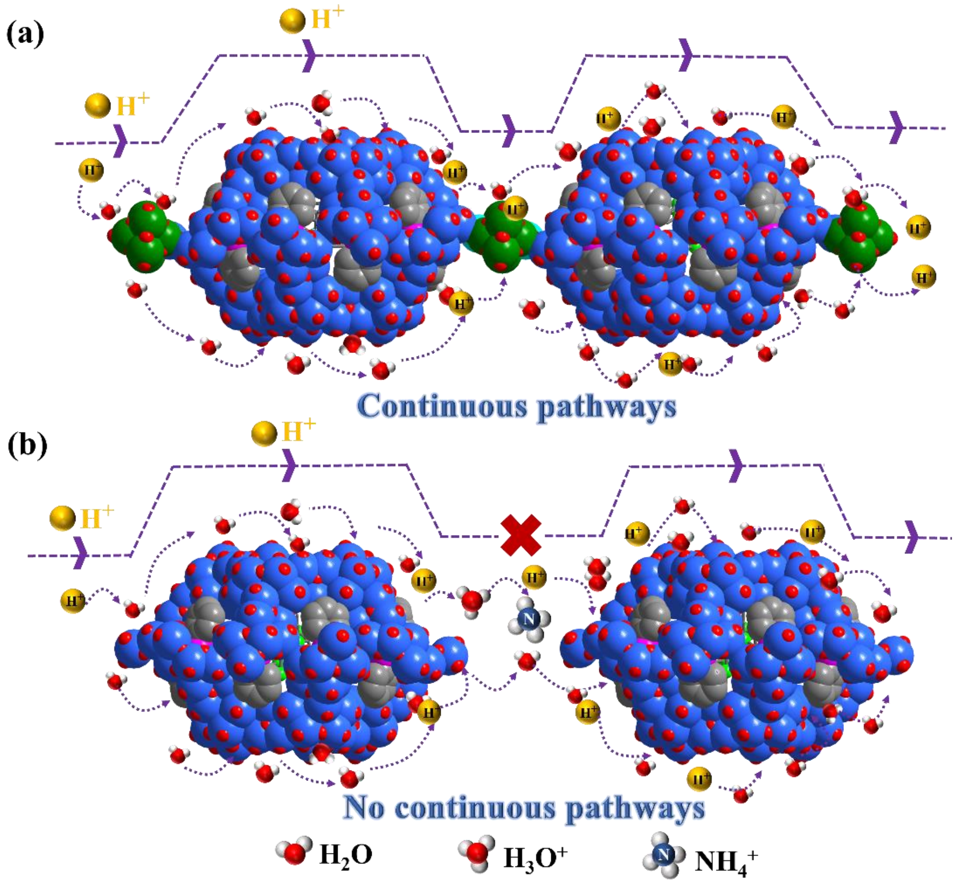

Figure S27. The proton transport mechanism of **1** and **2.**

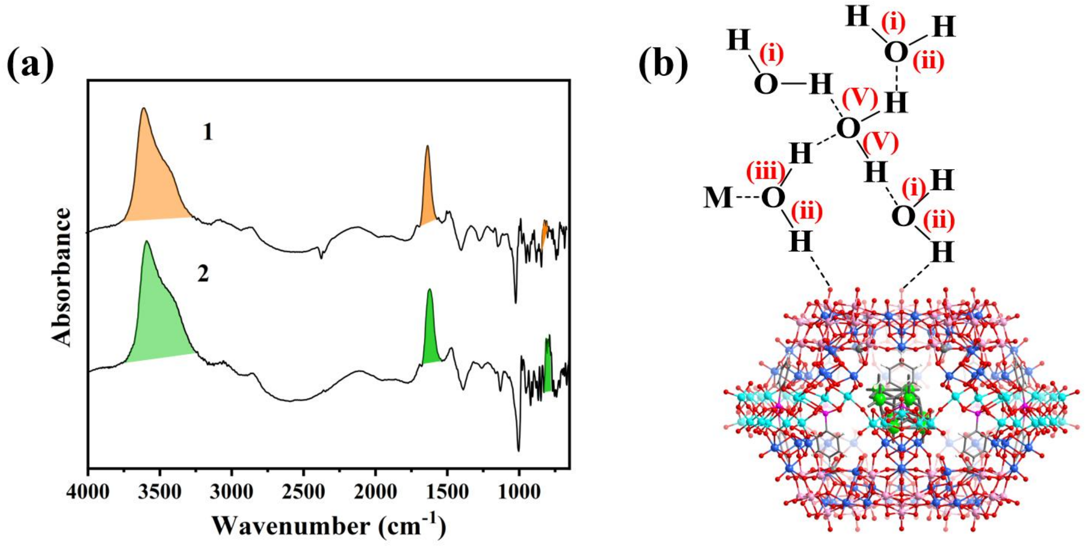

Figure S28. In-situ IR spectra (298 K) of **1** and **2** under a water vapor pressure of 0.1Mpa (P/P_0 = 0.98).

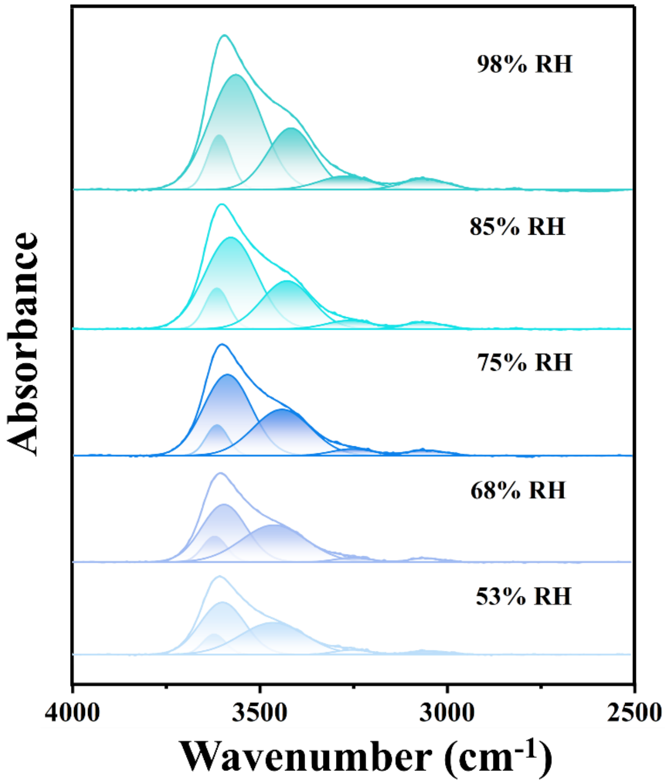

Figure S29. ATR-IR spectra of **1** in the OH stretch region at different RHs.

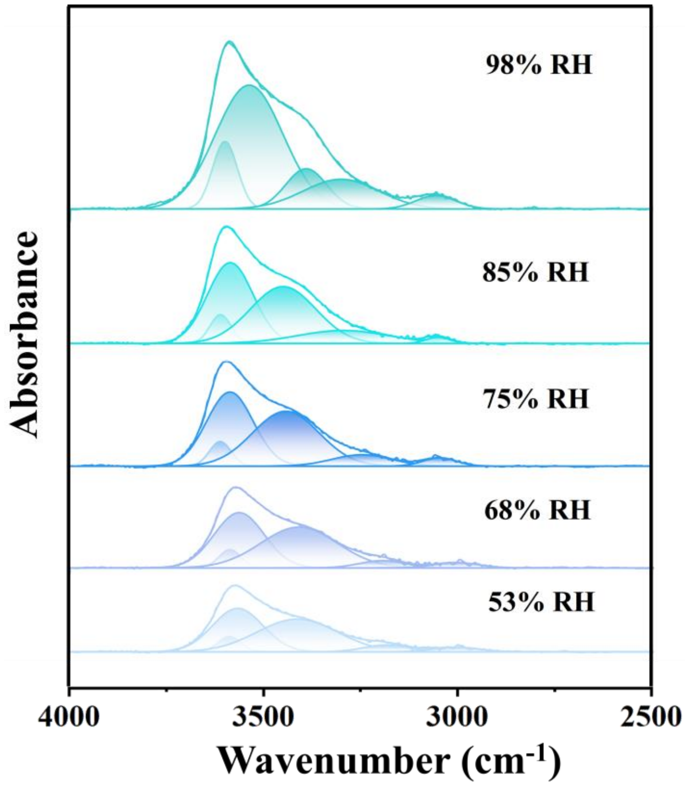

Figure S30. ATR-IR spectra of **2** in the OH stretch region at different RHs.

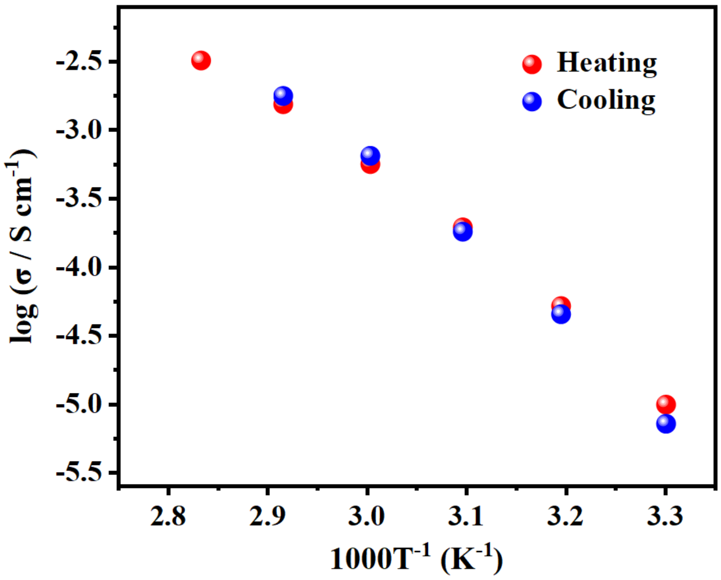

Figure S31. Arrhenius plots of the proton conductivity of **1** during one heating and cooling cycle at 98% RH.

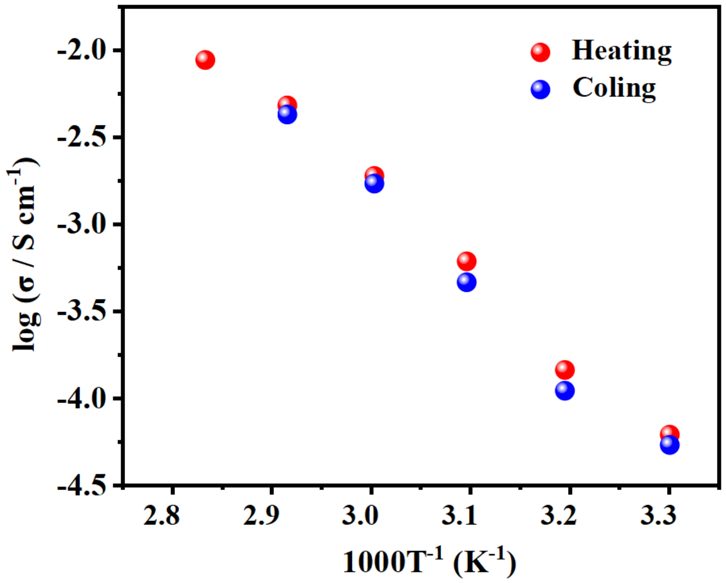

Figure S32. Arrhenius plots of the proton conductivity of **2** during one heating and cooling cycle at 98% RH.

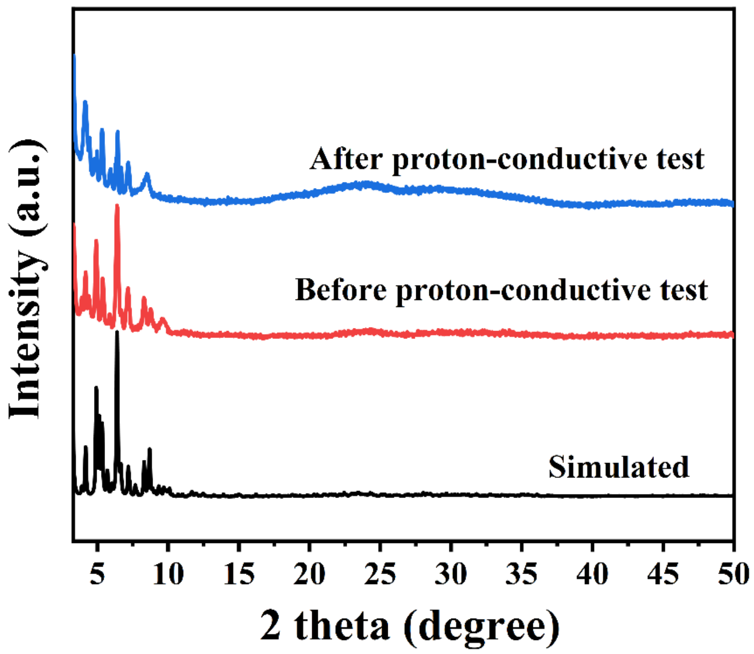

Figure S33. Powder XRD spectra of **1** before and after proton conductivity test.

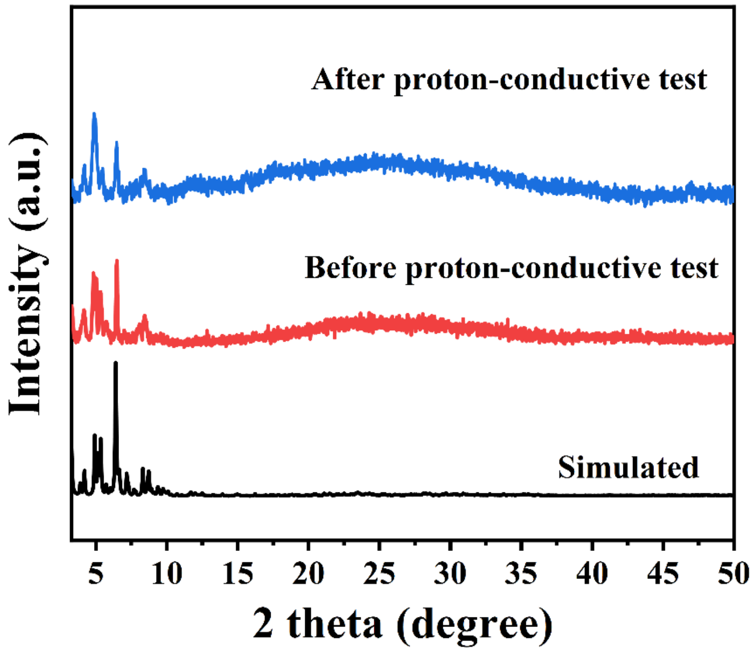

Figure S34. Powder XRD spectra of **2** before and after proton conductivity test.

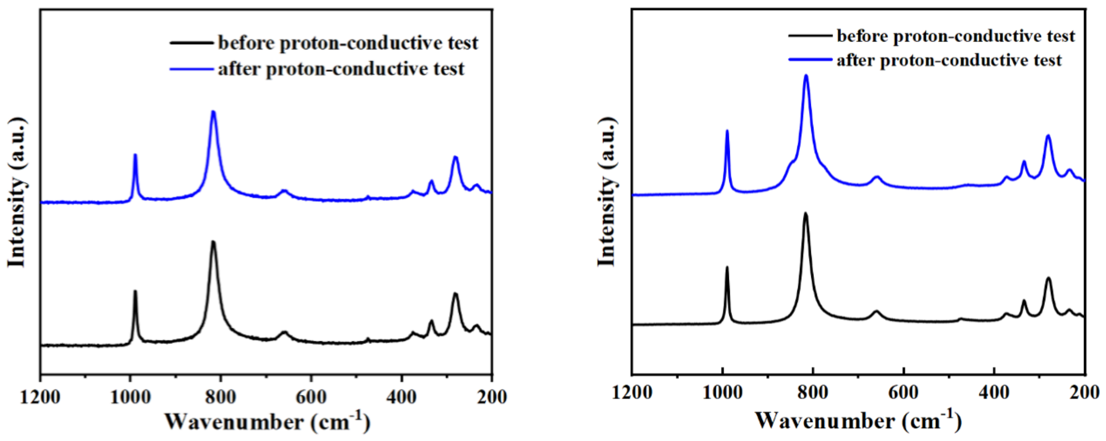

Figure S35. Raman spectra for **1** (left) and **2** (right) before and after proton-conductive measurement.

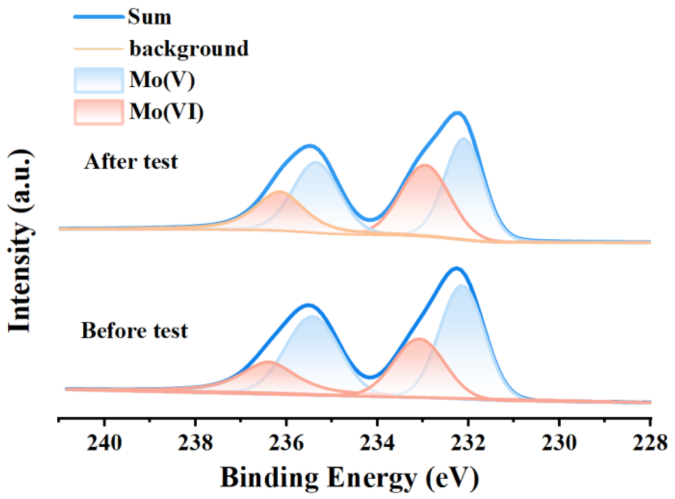

Figure S36. XPS spectra of Mo in **1** before and after proton-conductive measurement.

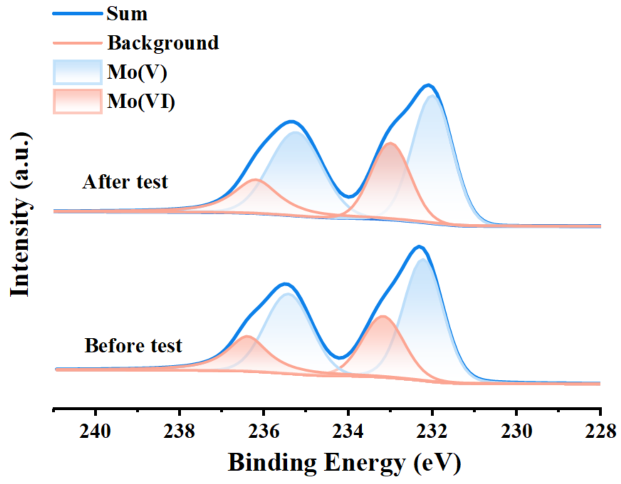

Figure S37. XPS spectra of Mo in **2** before and after proton-conductive measurement.

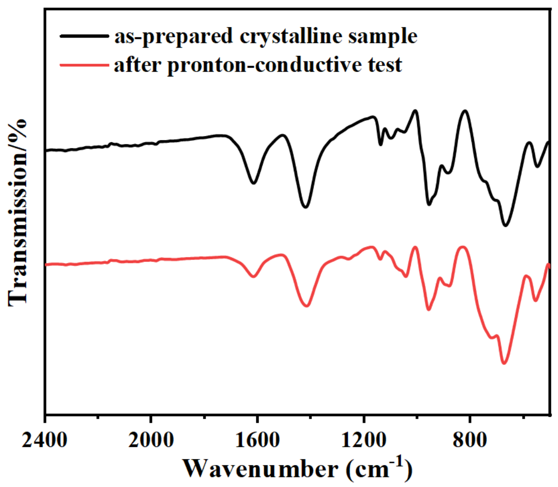

Figure S38. FT-IR spectra for **1** before (black) and after (red) proton-conductive measurement.

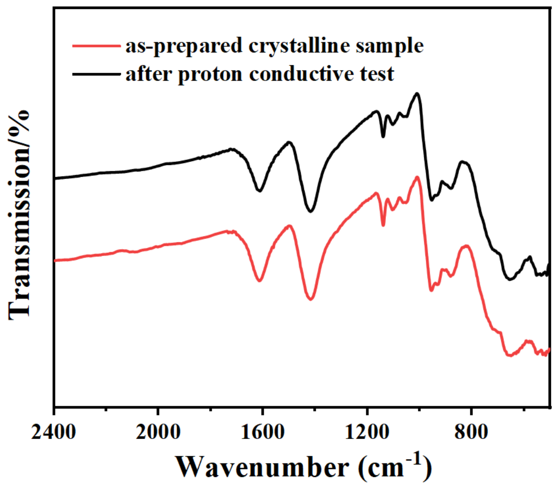

Figure S39. FT-IR spectra for **2** before (black) and after (red) proton-conductive measurement.

Table S7. Resistance (R) and proton conductivity (σ) of **2** under different relative humidities at 30°C

**RH (%)**	**R (Ω)**	**σ (S cm^{-1})**
53	905	6.75×10^{-4}
68	370	1.65×10^{-3}
75	131	4.67×10^{-3}
85	70	8.76×10^{-3}
98	41	1.49×10^{-2}

Table S8. Resistance (R) and proton conductivity (σ) of **2** under different temperatures at 98% RH

**T (°C)**	**R (Ω)**	**σ (S cm^{-1})**
30	41.2	1.49×10^{-2}
40	28.3	2.16×10^{-2}
50	15.2	4.03×10^{-2}
60	9.3	6.58×10^{-2}
70	6.2	9.86×10^{-2}
80	4.8	1.28×10^{-1}

Table S9. A comparison of the proton conductivity of **1-2** and some other recent representative POMs-based crystalline conducting materials.

**Year**	**POMs-based crystalline conducting materials**	**Proton conductivity (S cm^{-1})**	**Relative Humidity**	**Temperature (°C)**	***E*$_a$ (eV)**	***Reference***
-	**Compound 1**	6.72×10^{-3}	98%	30	0.38	**This work**
		8.28×10^{-2}	98%	80		
	**Compound 2**	1.49×10^{-2}	98%	30	0.36	
		1.28×10^{-1}	98%	80		
2024	$[Mo^{V}_{32}Mo^{VI}_{8}(OH)_{12}O_{10}]^{20-}$	8.16×10^{-4}	98%	25	0.27	8
		7.56×10^{-3}	98%	70		
	$[Mo^{V}_{24}Mo^{VI}_{6}(OH)_{10}O_{80}]^{14-}$	7.23×10^{-4}	98%	25	0.26	
		4.51×10^{-3}	98%	70		
	$[Mo^{V}_{16}Mo^{VI}_{12}O_{84}]^{16-}$	6.27×10^{-4}	98%	25	0.29	
		3.03×10^{-3}	98%	70		
2023	$[C_4H_6N_2]_2[Co(H_2O)_5][Co(H_2O)_2]_2\{Co[(PO_3)_3(PO_4)Mo_6O_{15}]_2\}^{14-}$	1.33×10^{-2}	98%	75	-	9
	$[C_4H_6N_2]_2[Fe(H_2O)_5][Fe(H_2O)_2]_2\{Fe[(PO_3)_3(PO_4)Mo_6O_{15}]_2\}^{14-}$	1.03×10^{-2}	98%	75	-	
2023	$[Ce_{11}Mo_{96}O_{286}(H_2O)_{101}(SO_4)_8]^{9-}$	2.50×10^{-2}	98%	30	0.26	10

		0.91×10^{-1}	98%	80		
2022	$[Cu(en)_2(H_2O)]_2[Cu(en)_2]_{10}H_{97}[Dy_{10}Nb_{190}]^{7-}$	1.19×10^{-4}	98%	25	0.54	11
		3.75×10^{-3}	98%	85		
2022	$[(AsW_9O_{33})_6\{W_2O_5(H_2O)(Ala)\}_2\{W_3O_6(H_2O)(Ala)\}_2\{W_2O_5(Ala)\}]$	2.83×10^{-4}	75%	65	0.54	12
2022	$H_4[Cu(en)_2]_4\{K_4(H_2O)_2[Cu(en)_2]_5[Cu_5(trz)_2(en)_4(OH)_2][Dy_2Cu_2(en)_2(CO_3)_3(H_2O)_2(OH)_3][Dy(H_2O)_4][DyNb_{23}O_{68}(H_2O)_4]_2\}\cdot 60H_2O$	4.68×10^{-6}	98%	25	1.03	13
		3.42×10^{-3}	98%	85		
2022	$[(NaP_5W_{30}O_{110})_2\subset Mo_{22}(Fe\text{-}edta)_8O_{68}(H_2O)_2]^{40-}$	1.7×10^{-2}	90%	95	0.31	14
2022	$[\{Mo_{24}O_{48}(OMe)_{32}\}\{Mo_{24}O_{52}(OMe)_{28}\}_2]^{8-}$	6.73×10^{-6}	98%	35	0.49	15
		1.79×10^{-3}	98%	85		
2022	$\{Ln_4(L)_2(H_2O)_{21}[Zr_3(OH)_3(PW_9O_{34})_2]\}^{1-}$	7.53×10^{-3}	98%	85	0.30	16
2022	$\{[Co(en)_2(SO_3)][Te_4Nb_{24}O_{79}]\}^{20-}$	8.13×10^{-5}	75%	25	0.28	17
		3.05×10^{-4}	75%	60		
2021	$\{[Cu(en)_2]_{10}[Nb_{68}O_{182}(OH)_8(H_2O)_{10}]\}^{12-}$	9.67×10^{-5}	98%	25	0.53	18
		5.71×10^{-3}	98%	75		
2021	$[Co(H_2O)_6]_2\{[Co(H_2O)_4]_4[WZn_3(H_2O)_2(ZnW_9O_{34})_2]\}$	3.55×10^{-4}	98%	85	0.24	19
2021	$\{[P_2W_{15}Nb_3O_{62}]_2(4PBA)_2((4PBA)_2O)\}^{16-}$	1.64×10^{-3}	98%	20	0.66	20
2020	$[Mo^{V}_{180}Mo^{VI}_{60}(OH)_{60}O_{620\text{-}x}(SO_3)_{20\text{-}x}(SO_4)_x]^{-(80\text{-}2x)}$	3.3×10^{-2}	98%	25	0.24	21
		1.03×10^{-1}	98%	80		
2019	$[Mo_{72}{}^{VI}Mo_{60}{}^{V}O_{372}(CH_3COO)_{30}(H_2O)_{72}]^{44-}$	6.2×10^{-3}	98%	25	0.51	22
		5.0×10^{-2}	98%	60		
2018	$\{[W_{14}Ce^{IV}{}_6O_{61}]([W_3Bi_6Ce^{III}{}_3(H_2O)_3O_{14}][BiW_9O_{33}]_3)_2\}^{34-}$	2.4×10^{-3}	90%	25	0.68	23
2018	$\{[Na(NO_3)(H_2O)]_4[Al_{16}(OH)_{24}(H_2O)_8(P_8W_{48}O_{184})]\}^{16-}$	9.1×10^{-3}	85%	25	0.32	24
		4.5×10^{-2}	70%	85		
2018	$[Na_6(H_2O)_{12}]_4[K_{42}Ge_8W_{72}O_{272}(H_2O)_{60}]^{14-}$	3.3×10^{-3}	98%	30	0.52	25
		6.8×10^{-2}	98%	80		
2018	$(1H\text{-}1,2,4\text{-}triazole)_8\{V_{10}\}$	1.24×10^{-2}	98	60	0.53	26
2018	$\{Bi_{18}Ce_{12}W_{74}\}$	2.4×10^{-3}	90	25	0.68	23
2017	$[La_{27}Ge_{10}W_{106}O_{406}(OH)_4(H_2O)_{24}]^{59-}$	4.0×10^{-5}	98%	30	0.42	27
		1.5×10^{-2}	98%	85		
2016	$[Ce^{III}(H_2O)_6]\{[Ce^{IV}{}_7Ce^{III}{}_3O_6(OH)_6(CO_3)(H_2O)_{11}][(P_2W_{16}O_{59})]_3\}^{16-}$	1.95×10^{-7}	75%	30	0.36	28
		2.65×10^{-4}	75%	100		

12. References

1. Sheldrick, G. Crystal structure refinement with SHELXL. *Acta Crystallogr C* **2015**, 71, 3-8.

2. Sheldrick, G. M. SHELXT-Integrated space-group and crystal-structure determination. *Acta Crystallogr. A: Found. Adv.* **2015,** 71, 3-8.

3. Dolomanov, O. V.; Bourhis, L. J.; Gildea, R. J.; Howard, J. A.; Puschmann, H. OLEX2: a complete structure solution, refinement and analysis program. *J. Appl. Crystallogr.* **2009**, 42, 339-341.

4. Rees, B.; Jenner, L.; Yusupov, M. Bulk-solvent correction in large macromolecular structures. *Acta Crystallogr. D: Biol. Crystallogr.* **2005,** 61, 1299-1301.

5. Gagné, O. C.; Hawthorne, F. C. Comprehensive derivation of bond-valence parameters for ion pairs involving oxygen. *Acta. Crystallogr., Sect. B: Struct. Sci., Cryst. Eng. and Mater.* **2015,** 71, 562-578.

6. Atovmyan, L.; Krasochka, O. X-ray diffraction investigation of the crystals of the octamolybdate $(NH_4)_4Mo_8O_{26}\cdot 4H_2O$. *J. Struct. Chem.* **1972**, 13, 319-320.

7. Dong, B.; Xu, Q. Investigation of flexible organic ligands in the molybdate system: delicate influence of a peripheral cluster environment on the isopolymolybdate frameworks. *Inorg. Chem.* **2009**, 48, 5861-5873.

8. Li, B.; Lan, Y.; Su, H.; Xu, J.; Zhao, Q.; Ma, Y.; Zheng, Q.; Xuan, W. $\{Mo_4\}$-directed structural evolution of highly reduced molybdenum red clusters for efficient proton conduction. *Dalton Trans.* **2024,** 53, 6184-6189.

9. Gao, Z.-X.; Sun, S.; Li, B.; Cheng, D.-M.; Wang, Y.-H.; Zang, H.-Y.; Li, Y.-G. Design and synthesis of phosphomolybdate coordination compounds based on $\{P_4Mo_6\}$ structural units and their proton conductivity. *Tungsten* **2023,** 1-8.

10. Li, X.-X.; Li, C.-H.; Hou, M.-J.; Zhu, B.; Chen, W.-C.; Sun, C.-Y.; Yuan, Y.; Guan, W.; Qin, C.; Shao, K.-Z. Ce-mediated molecular tailoring on gigantic polyoxometalate $\{Mo_{132}\}$ into half-closed $\{Ce_{11}Mo_{96}\}$ for high proton conduction. *Nat. Commun*. **2023,** 14, 5025.

11. Lai, R.-D.; Zhang, J.; Li, X.-X.; Zheng, S.-T.; Yang, G.-Y. Assemblies of increasingly large Ln-containing polyoxoniobates and intermolecular aggregation–disaggregation interconversions. *J. Am. Chem. Soc.* **2022,** 144, 19603-19610.

12. Zheng, K.; Yang, D.; Niu, B.; Ye, Y.; Ma, P.; Wang, J.; Niu, J. DL-Alanine covalently bonded giant arsenotungstate with rapid photochromic and decent proton conduction properties. *Inorg. Chem.* **2022,** 61, 20222-20226.

13. Lai, R.-D.; Zhu, Z.-K.; Wu, Y.-L.; Sun, Y.-Q.; Sun, C.; Li, X.-X.; Zheng, S.-T. Proton-Conductive Polyoxometalate Architectures Constructed from Lanthanide-Incorporated Polyoxoniobate Cages. *Inorg. Chem.* **2022,** 61, 21047-21054.

14. Zhu, M.; Iwano, T.; Tan, M.; Akutsu, D.; Uchida, S.; Chen, G.; Fang, X. Macrocyclic polyoxometalates: selective polyanion binding and ultrahigh proton conduction. *Angew. Chem., Int. Ed.* **2022,** 61, e202200666.

15. Wang, Y.; Ma, X.; Li, G.; Li, H.; Wang, Q.; Chen, W.; Ma, P.; Li, S.; Niu, J.; Wang, J. A High-Nuclear Isopolymolybdate Cluster Assembled with an Anionic $[\{Mo_{24}O_{48}(OMe)_{32}\}]^{8-}$ and Two Charge-Neutral $[\{Mo_{24}O_{52}(OMe)_{28}\}]$ Cages. *Chem. Eur. J.* **2022,** 28, e202200637.

16. Fan, Y.-H.; Du, M.; Li, Y.-X.; Zhu, W.-J.; Pang, J.-Y.; Bai, Y.; Dang, D.-B. Construction of water-stable rare-earth organic frameworks with ambient high proton conductivity based on zirconium sandwiched heteropolytungstate. *Inorg. Chem.* **2022,** 61, 13829-13835.

17. Jing, Z.; Wang, W.; Wan, R.; Ma, X.; Qiao, Y.; Bai, Y.; Ma, P.; Niu, J.; Wang, J. Rocket-Shaped

Telluroniobate with Efficient Catalytic Activity in the Transesterification Reaction. *Inorg. Chem.* **2022,** 61, 16528-16532.
18. Lin, Y.-D.; Zhu, Z.-K.; Ge, R.; Yu, H.; Li, Z.; Sun, C.; Sun, Y.-Q.; Li, X.-X.; Zheng, S.-T. Proton conductive polyoxoniobate frameworks constructed from nanoscale {$Nb_{68}O_{200}$} cages. *Chem. Commun.* **2021,** 57, 4702-4705.
19. Si, C.; Ma, P.; Han, Q.; Jiao, J.; Du, W.; Wu, J.; Li, M.; Niu, J. A Polyoxometalate-Based Inorganic Porous Material with both Proton and Electron Conductivity by Light Actuation: Photocatalysis for Baeyer–Villiger Oxidation and Cr (VI) Reduction. *Inorg. Chem.* **2020,** 60, 682-691.
20. Li, S.; Zhao, Y.; Knoll, S.; Liu, R.; Li, G.; Peng, Q.; Qiu, P.; He, D.; Streb, C.; Chen, X. High proton-conductivity in covalently linked polyoxometalate-organoboronic acid-polymers. *Angew. Chem., Int. Ed.* **2021,** 60, 16953-16957.
21. Lin, J.; Li, N.; Yang, S.; Jia, M.; Liu, J.; Li, X.-M.; An, L.; Tian, Q.; Dong, L.-Z.; Lan, Y.-Q. Self-assembly of giant Mo_{240} hollow opening dodecahedra. *J. Am. Chem. Soc.* **2020,** 142, 13982-13988.
22. Liu, W.-J.; Dong, L.-Z.; Li, R.-H.; Chen, Y.-J.; Sun, S.-N.; Li, S.-L.; Lan, Y.-Q. Different protonic species affecting proton conductivity in hollow spherelike polyoxometalates. *ACS Appl. Mater. Interfaces* **2019,** 11, 7030-7036.
23. Liu, J. C.; Han, Q.; Chen, L. J.; Zhao, J. W.; Streb, C.; Song, Y. F. Aggregation of giant cerium–bismuth tungstate clusters into a 3D porous framework with high proton conductivity. *Angew. Chem., Int. Ed.* **2018,** 57, 8416-8420.
24. Yang, P.; Alsufyani, M.; Emwas, A. H.; Chen, C.; Khashab, N. M. Lewis Acid Guests in a {P_8W_{48}} Archetypal Polyoxotungstate Host: Enhanced Proton Conductivity via Metal-Oxo Cluster within Cluster Assemblies. *Angew. Chem., Int. Ed.* **2018,** 130, 13230-13235.
25. Li, Z.; Lin, L. D.; Yu, H.; Li, X. X.; Zheng, S. T. All-Inorganic Ionic Porous Material Based on Giant Spherical Polyoxometalates Containing Core-Shell K_6@K_{36}-Water Cage. *Angew. Chem., Int. Ed.* **2018,** 130, 16003-16007.
26. Cao, J. P.; Shen, F.-C.; Luo, X.-M.; Cui, C.-H.; Lan, Y.-Q.; Xu, Y. Proton conductivity resulting from different triazole-based ligands in two new bifunctional decavanadates. *RSC advances* **2018,** 8, 18560-18566.
27. Li, Z.; Li, X. X.; Yang, T.; Cai, Z. W.; Zheng, S. T. Four-Shell Polyoxometalates Featuring High-Nuclearity Ln_{26} Clusters: Structural Transformations of Nanoclusters into Frameworks Triggered by Transition-Metal Ions. *Angew. Chem., Int. Ed.* **2017,** 129, 2708-2713.
28. Ma, P.; Wan, R.; Wang, Y.; Hu, F.; Zhang, D.; Niu, J.; Wang, J. Coordination-driven self-assembly of a 2D graphite-like framework constructed from high-nuclear Ce_{10} cluster encapsulated polyoxotungstates. *Inorg. Chem.* **2016,** 55, 918-924.
